# Scavenger receptors: key players in the immunological puzzle of lupus

**DOI:** 10.3389/flupu.2025.1679564

**Published:** 2025-10-28

**Authors:** Sabine Hahn, Monika Chitre, Dominique Shepard, Romana Rashid, Zaida G. Ramirez-Ortiz

**Affiliations:** Department of Medicine, Division of Infectious Disease and Immunology, University of Massachusetts Chan Medical School, Worcester, MA, United States

**Keywords:** scavenger receptors (SRs), autoimmunity, systemic lupus erythematosus (SLE), inflammation, cardinal signs

## Abstract

Scavenger receptors (SRs) play an important role in the innate immune response by recognizing and binding a variety of ligands to initiate the removal of both altered self- and non-self-antigens. Over the last two decades, SRs have become a forefront for their role influencing and contributing to inflammatory disease pathways. The findings discussed in this review show that the immunological role SRs play is (1) found in multiple organ systems and not limited to one disease or subset of symptoms; (2) part of both the innate and adaptive immune response in addition to influencing inflammatory signaling via non-immune cell subtypes; (3) both pro- or anti-inflammatory depending on which SR class or cell signaling pathway is being observed; (4) potentially useful for the development of therapeutics and diagnostic or prognostic biomarkers for autoimmune disease pathology. Understanding the role of SRs in the context of inflammation and autoimmunity will shed some light on the comprehension of heterogeneous diseases, such as Systemic Lupus Erythematosus.

## 1 Introduction

**FIGURE 1:**
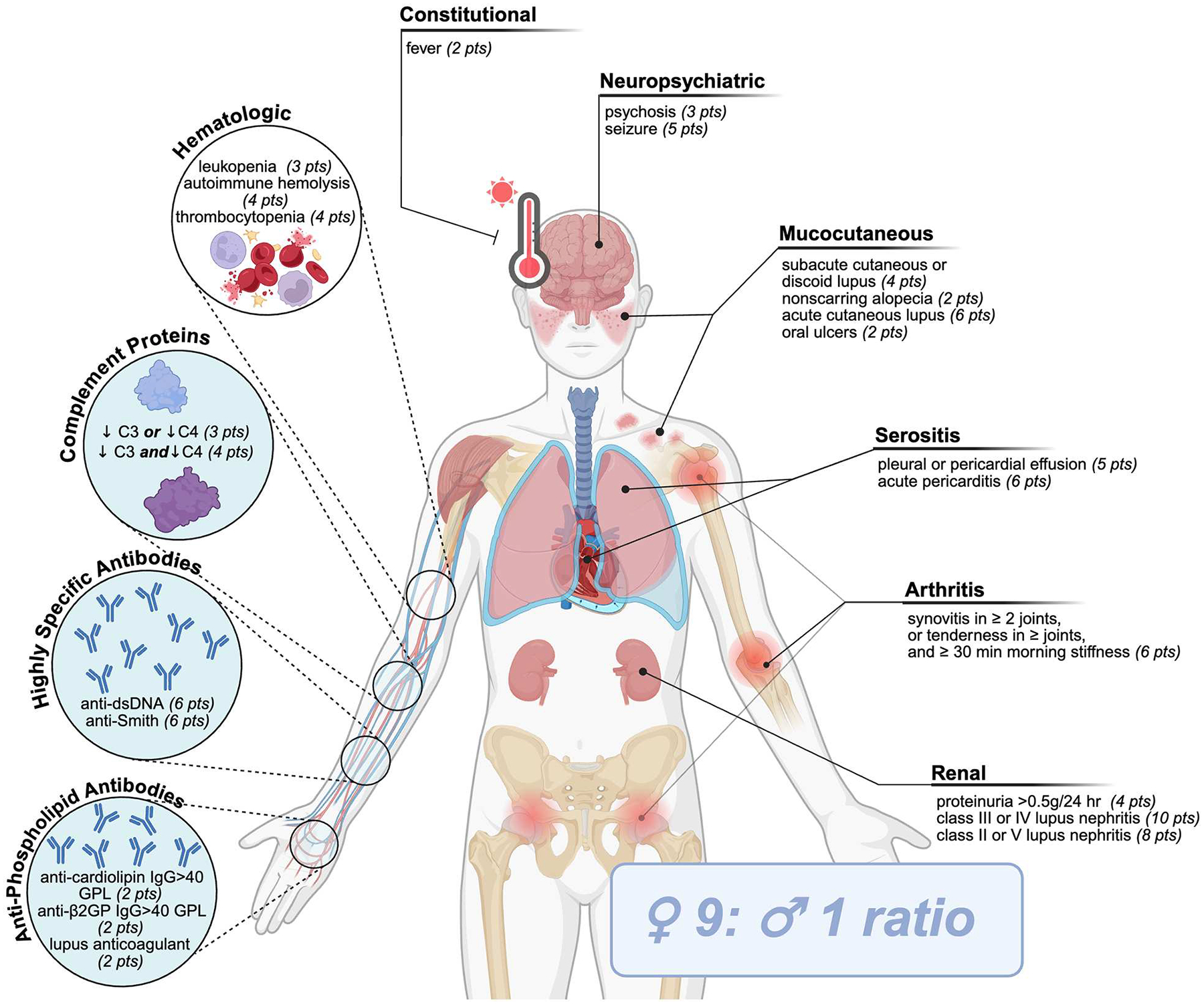
SLE classification criteria. Current criteria (2019 European League Against Rheumatism/American College of Rheumatology Immunologic Domains and Criteria for SLE) requires patients to first test positive for antinuclear antibodies, followed by an assessment of a combination of 10 clinical (uncolored) or immunological (blue circles) symptoms or disorders, each weighted differently to calculate a final score amounting to ≥10 points.

Systemic lupus erythematosus (SLE) is a complex and multifaceted autoimmune disease. It is represented by diverse clinical features impacting multiple organs, combined with a wide range of hematological and serological abnormalities, and disease comorbidities (1). The current classification criteria [2019 European League Against Rheumatism/American College of Rheumatology Immunologic Domains and Criteria for SLE (2019 EULAR/ACR)] requires patients to first test positive for antinuclear antibodies and is followed by an assessment of a combination of 10 symptoms or disorders, each weighted differently to calculate a final score (Figure 1) (2, 3). Many genetic deficiencies are associated with the development of SLE; however, most patients inherit the disease polygenically in conjunction with environmental, hormonal, or co-infectious triggers (1, 4, 5). The immunological defects leading to the development of SLE are a product of two stages: first, systemic autoimmunity that induces serum autoantibodies and, second, immunological events that induce organ damage (6). Immune dysregulation in both innate and adaptive immune systems can impact the activation and progression of SLE (1, 6). Nonetheless, diagnosis and subsequent treatment of SLE have been hampered due to heterogenic clinicopathological presentation of the disease (1, 4, 7).

Immune homeostasis is vital for the balance of immune activation and suppression across tissues and organs to prevent damage due to excessive, malfunctioning, and/or self-targeting immune responses (8). The scavenger receptors (SRs) superfamily (Table 1) contributes to maintaining homeostasis through their roles- within cellular transport, nutrient exchange, and waste removal as well as their roles directly within immunity through the identification and presentation of antigens, regulation of inflammation, and adhesion of leukocytes (9, 10). SRs act as cell surface receptors, but can also be found intracellularly or as soluble forms within circulation where they can bind and promote the removal of an array of unwanted ligands—either self or non-self—via endocytosis, phagocytosis, and micropinocytosis (9, 11). Examples of these ligands include apoptotic cells, damage proteins, and heat shock proteins. Beyond scavenging for waste products to maintain homeostasis, SRs simultaneously help conserve the appropriate balance of endogenous molecules, identify damaged antigens, and elicit the proper immune responses (9). Over the past 40 years, numerous SRs have been identified. This superfamily of receptors has been divided into 10 distinct classes (12) based on their nucleotide sequence alignment and protein structure, with each class further divided into subclasses that share specific structural features (Supplementary Table 1) (11, 13, 14). However, as the field is evolving, some newly uncovered SRs remain unclassified while others are classified as noncanonical SRs, or proteins that simply exhibit scavenger receptor activity (12). Several SRs have been implicated in the progression of autoimmune diseases, including SLE, through mechanisms including inflammation, apoptosis, foam cell formation, T cell activation, and the complement pathway. This review will discuss the SRs identified to date that play a role in autoinflammation and autoimmunity, particularly in the context of SLE.

## 2 Scavenger receptors in clinical hallmarks of SLE inflammation

**FIGURE 2:**
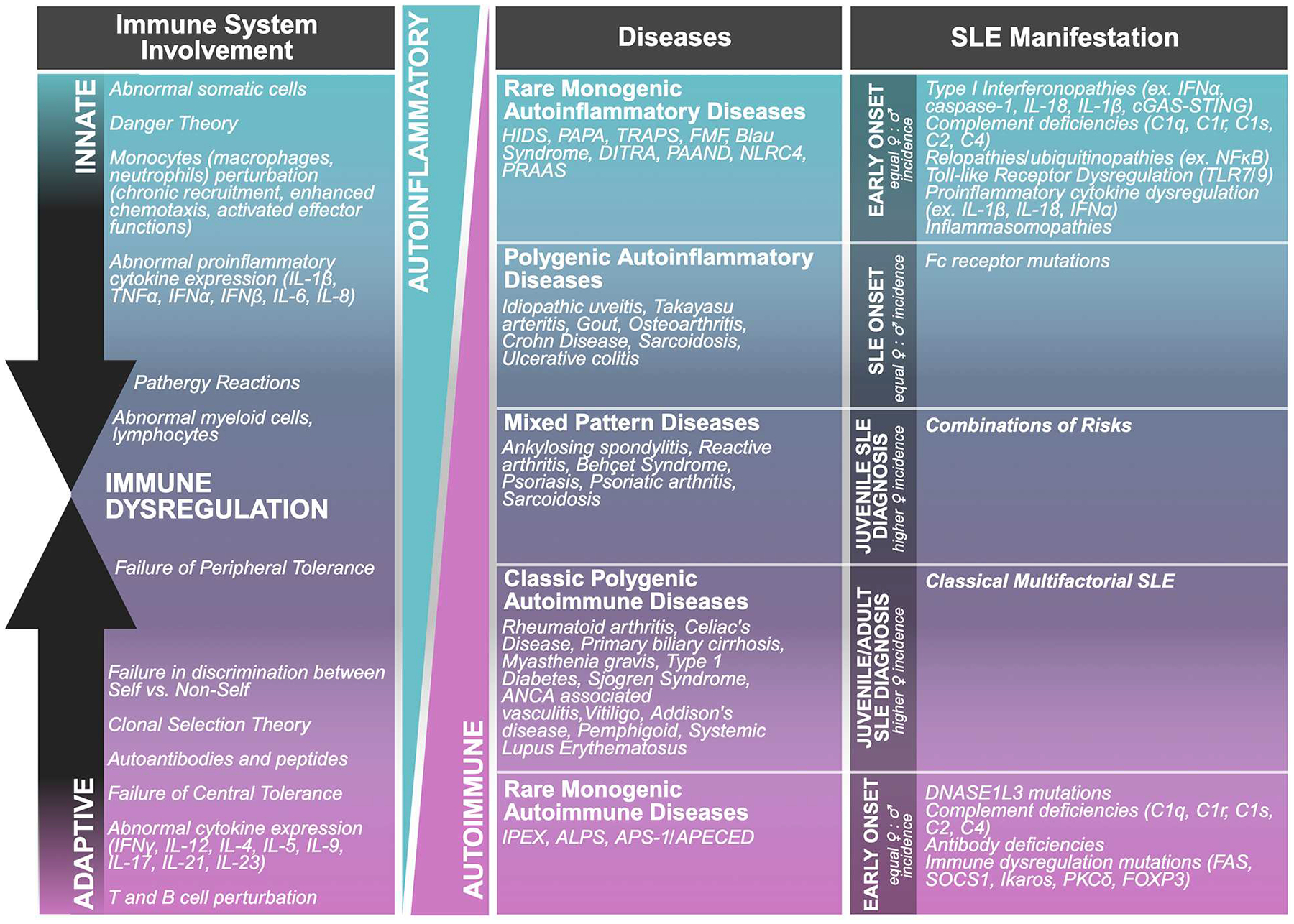
Immunological disease continuum model. This model proposes a spectrum of immune-mediated diseases that ranges from autoinflammatory to autoimmune condition.

**FIGURE 3:**
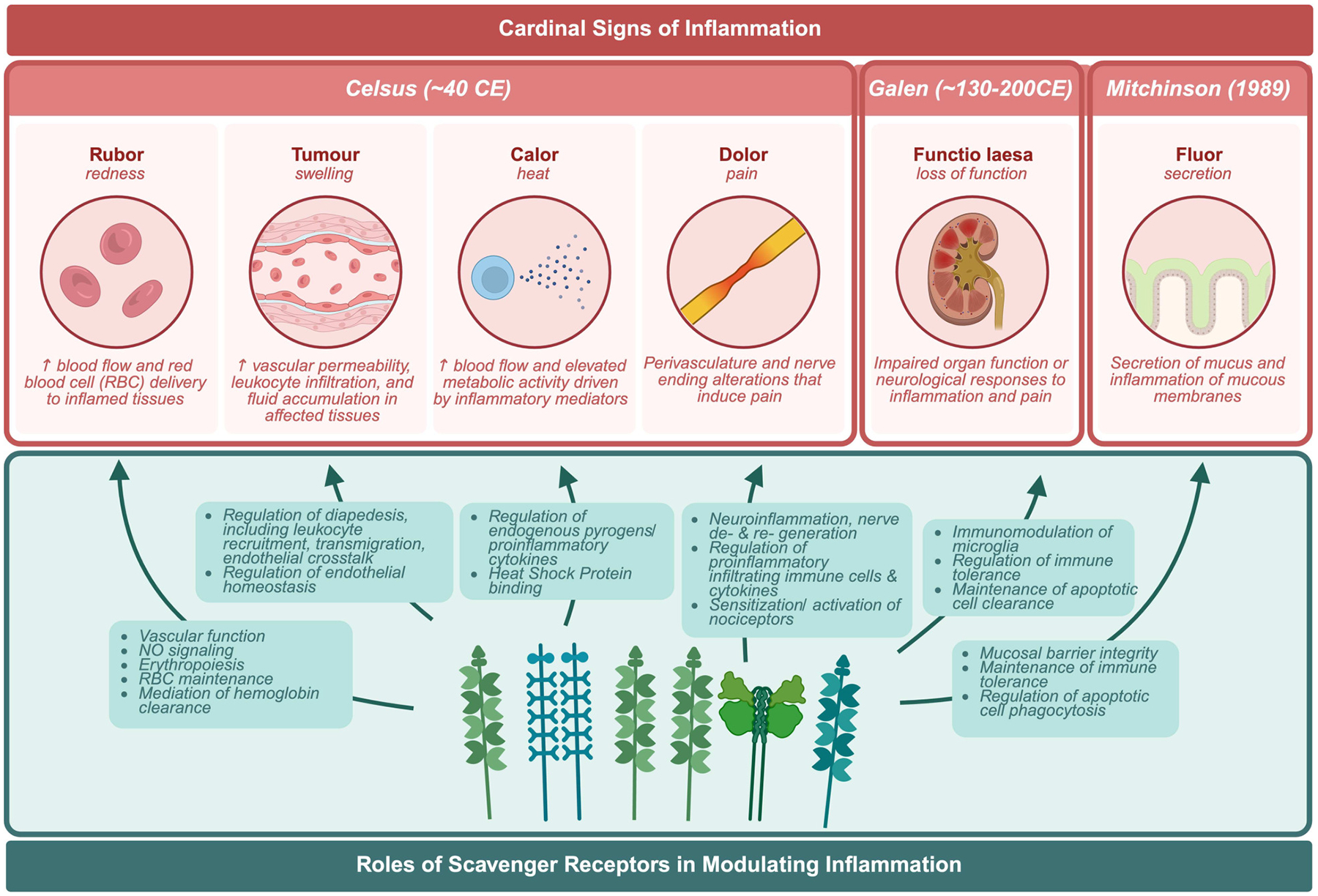
Cardinal signs of inflammation. Biological responses of the body to harmful stimuli in response to scavenger receptors.

Nearly twenty years ago an “immunological disease continuum model” was proposed by McGonagle and Dermott (15). The model suggests that noninfectious diseases lie on a spectrum from autoimmune to autoinflammatory, thereby classifying the roles of both the innate and adaptive immune responses in immunological disease, contextualizing noninfectious diseases (Figure 2). Ultimately, the model enhanced our understanding of the pathogenesis and treatment of immune reactivity against self, or as Paul Ehrlich himself called it, “*horror autotoxicus*” (15), the development of immune reactivity to self and loss of immune tolerance through aberrant dendritic, B, and T cell responses (15, 16). However, placing immunological diseases like SLE along this continuum provides an alternative perspective on the heterogeneity of clinical disease manifestation through the contribution of different inflammatory responses, without assuming that the adaptive immune response is central to disease pathogenesis. For the development of therapies, autoimmune diseases with autoinflammatory components could potentially benefit from innate immune pathway targeting (15, 16). Patients with SLE can experience inflammation in any organ (17). Local signs and symptoms of clinical inflammation were originally described by Celsus in the first century BC as *rubor* (redness), *tumour* (swelling), *calor* (heat), and *dolor* (pain) (Figure 3). A century later Galen added *functio laesa* (loss of function) and nearly two thousand years later, Mitchinson added a sixth, *fluor* (secretion) (18–21). These clinical hallmarks of inflammation may be inherently compromised or chronically unresolved in patients with SLE (18, 20).

### 2.1 Rubor

*Rubor*, or redness, is one of the classical signs of inflammation, arising from hyperemia—an increase in blood flow and red blood cell (RBC) delivery to inflamed tissues (22). SRs, initially characterized by their role in binding modified low- and high-density lipoproteins (LDL and HDL), have since been found to play multifaceted roles in vascular biology. They regulate endothelial integrity, vasodilation, red blood cell homeostasis, and immune responses, making them central to the inflammatory and vascular manifestations of autoimmune diseases such as SLE (23–25).

#### 2.1.1 Rubor: vasculature & vasodilation

Scavenger receptor class B member 1 (SR-B1) is essential for lipid and cholesterol metabolism as well as vascular function. Expressed on endothelial cells, vascular smooth muscle cells, monocytes, and macrophages, SR-B1 facilitates HDL binding and mediates endothelium-dependent vasodilation, primarily through the synthesis and activity of nitric oxide (NO) (26–32). NO is a key signaling molecule in vascular homeostasis, responsible for modulating vasodilation, inhibiting leukocyte adhesion, and regulating platelet activity (33). *In vivo* studies demonstrate that SR-B1 deficiency leads to significant vascular dysfunction, including impaired NO signaling, dyslipidemia, increased platelet aggregation, and development of atherosclerotic lesions (26, 28–31) (34). For example, mice lacking SR-B1 exhibit thrombocytopenia, thrombomegaly, and heightened susceptibility to thrombotic events (34, 35). These symptoms are observed in 20%–40% of SLE patients. Moreover, SLE is associated with elevated systemic NO levels, suggesting that NO dysregulation may serve as a biomarker for disease activity (36, 37). Further, loss of SR-B1 *in vivo* contributes to coronary artery disease, myocardial infarction, ischemic cardiomyopathy, and heart failure—all of which are significantly more prevalent in SLE patients (5, 24–26, 38–41). Accelerated atherosclerosis remains a leading cause of morbidity and mortality in SLE patients (5, 24, 25, 40, 41). In Takayasu arteritis, a type of autoimmune vasculitis, autoantibodies against SR-B1 interfere with HDL uptake and suppress nitric oxide synthase (NOS) activity, promoting endothelial inflammation and vascular damage (42). Similarly, SR-B1 has been implicated in the pathogenesis of other autoimmune diseases (43) and is strongly linked to cardiovascular disease (CVD), a common comorbidity in SLE (44). SLE patients have a higher risk of developing cardiovascular disease (CVD) (23–25) as well as increased risk for a broad spectrum of cardiovascular complications, including aortic wall inflammation (45), development of atherosclerosis (46), peripheral arterial disease (47), dyslipidemia (48, 49), heart failure (50), angina pectoris (51), and myocardial infarction (51, 52).

#### 2.1.2 Rubor: red blood cell development & maintenance

In addition to its vascular roles, SR-B1 also supports erythropoiesis and RBC maintenance (43). In SLE, RBCs frequently display abnormal morphology or size variability, which may impair oxygen delivery and contribute to common symptoms such as chronic fatigue, anemia, and cognitive dysfunction (53, 54). Autoimmune hemolytic anemia is another hematologic manifestation of SLE, leading to increased RBC destruction and free hemoglobin in circulation (55). SR-I1 or commonly known as CD163, is a hemoglobin-scavenging receptor primarily expressed on M2-polarized macrophages. It binds to haptoglobin–hemoglobin complexes to mediate hemoglobin clearance during intravascular hemolysis (55–58). In lupus nephritis (LN), kidney CD163^+^ macrophages infiltrate glomeruli, contributing to local inflammation and tissue damage (56, 59). Elevated levels of soluble SR-I1 are consistently found in the serum of patients with SLE, particularly those with macrophage activation syndrome, autoimmune hemolytic anemia, or immune thrombocytopenia (57, 60). Inflammatory conditions, including SLE, are characterized by increased expression of SR-I1, reflecting ongoing macrophage activation (55–57, 61).

Macrophage activation syndrome (MAS) is one of the most severe hyperinflammatory complications of lupus, characterized by uncontrolled macrophage and T cell activation, cytokine storm, and hemophagocytosis. While vascular and hematologic manifestations are well-established in SLE, defective SR pathways contributing to MAS susceptibility directly are limited (62, 63). In SLE, defective clearance of apoptotic debris [and release of nuclear danger/damage-associated molecular patterns (DAMPs) such as HMGB1] sustains TLR7/9 signaling and type I interferon production, which foster cytotoxic T-cell activation and excessive IFN- γ response—features characteristic of MAS (64–66). Under these inflammatory conditions, regulatory macrophage circuits such as the CD163-heme oxygenase-1 (HO-1) pathway, normally critical for heme detoxification and resolution of inflammation, may become functionally exhausted. Failure of this axis limits macrophage antioxidant and anti-inflammatory capacity and favors uncontrolled activation and hemophagocytosis, thereby lowering the threshold for MAS in SLE patients (67, 68). Serum soluble SR-I1 is consistently elevated in active MAS even under IL-6 blockade and closely reflects disease activity and the clinical relevance of SR dependent macrophage dysregulation (69, 70). Additional insights into SRs dysfunction and MAS pathogenesis emerge from related hyperinflammatory disorders. For example, in systemic juvenile idiopathic arthritis (sJIA), a disease with high MAS propensity, soluble SR-I1 correlates with MAS activity and TRIM8 upregulation in monocytes/macrophages augmenting IFN- γ responsiveness, providing a molecular mechanism for macrophage hyperactivation (70, 71).

Despite observed elevations in SR-I1, its precise role in SLE pathophysiology remains unclear. Two opposing hypotheses currently exist: the first suggests that SR-I1 is abnormally elevated, causing excessive phagocytosis of haptoglobin–hemoglobin complexes and downstream accumulation of iron, oxidative stress, and ferroptosis; the second proposes that SR-I1 is elevated as an adaptive response to prevent further tissue damage caused by ruptured red blood cells (55). Notably, soluble SR-I1 levels correlate with disease activity in rheumatoid arthritis (72) and serve as a biomarker for active renal involvement in SLE, including in the urine of patients with LN (73–75). Together, these findings underscore the dual vascular and hematologic roles of SRs such as SR-B1 and SR-I1 in the manifestation of *rubor* and broader SLE pathology. Their dysregulation contributes not only to visible signs of inflammation but also to systemic complications, making them potential biomarkers and therapeutic targets.

### 2.2 Calor

*Calor*, or heat, is a hallmark of inflammation and reflects increased blood flow and elevated metabolic activity driven by inflammatory mediators. During infection, fever (hyperthermia) is a protective physiological response that enhances immune function, promotes leukocyte trafficking, and inhibits pathogen replication (76–78). Historically, controlled elevation of body temperature—*therapeutic hyperthermia*—has even been employed to treat infectious disease by enhancing organ perfusion and activating immune pathways (76–78). However, excessive or sustained fever can lead to tissue damage and adverse outcomes, such as heat stroke (76).

In autoimmune diseases such as SLE, fever is a common manifestation—even in the absence of infection. Recurrent, unexplained fever is often a presenting symptom and is considered a clinical clue in early diagnosis (79–81). Studies report fever in 36%–86% of SLE patients, although the prevalence has declined due to routine use of nonsteroidal anti-inflammatory drugs (NSAIDs) (79, 81–86). Distinguishing SLE-related fever from infection remains clinically challenging, particularly in patients receiving immunosuppressive therapies that increase infection risk or due to inherent disease-associated immune perturbations (87, 88). In cases of Fever of Unknown Origin (FUO), infection must be rigorously ruled out before attributing symptoms to SLE (79). While infection is the most common cause of FUO, approximately 5% of cases are ultimately diagnosed as autoimmune conditions, including SLE and autoimmune thyroiditis (89, 90). Although SRs have not been directly linked to the development of fever, they influence proinflammatory signaling pathways, such as nuclear factor kappa-light-chain-enhancer of activated B cells (NF-κB) and JAK/STAT, that regulate the febrile response (383).

#### 2.2.1 Calor: endogenous pyrogens/cytokines

Fever is largely mediated by endogenous pyrogens—proinflammatory cytokines that are released by immune cells in response to infection, tissue damage, or autoimmune activity. Key endogenous pyrogens include tumor necrosis factor-α (TNF-α), interleukins (IL-1β, IL-6, IL-8), and interferons (IFN-β, IFN-γ), among others (91, 92). These cytokines circulate systemically and act on the hypothalamus to elevate body temperature. Many of these same cytokines are found at abnormally high levels in SLE patients, and their activity contributes to immune dysregulation, tissue damage, and systemic inflammation (93–100).

Due to this, they are also considered potential targets for SLE treatments (101). Scavenger receptor class E member 1 (SR-E1), also known as LOX-1, is a receptor for oxidized low-density lipoproteins (oxLDL) that is primarily expressed on vascular endothelial cells (102, 103), but also on vascular smooth muscle and lymphoid cells (104). SR-E1 mediates oxLDL endocytosis and promotes atherogenesis (104, 105) and has been shown to be a key player in the development of atherosclerosis, myocardial ischemia, hypertension, and inflammation (105, 106). SR-E1 deficiency *in vivo* protects mice from developing atherosclerosis (102), whereas SR-E1 overexpression promotes it (107). In SLE patients, SR-E1 expression is elevated even in early disease onset and low disease activity and correlates with high-sensitivity C-reactive protein (hsCRP), proinflammatory HDL, and oxLDL (108). Moreover, increased SR-E1 in SLE is associated with elevated IL-8 and reduced IFN-γ levels, while other cytokines such as IL-6, IL-10, and TNF-α remain unchanged (105). Interestingly, proinflammatory cytokines (e.g., IL-1β, TNF-α) can themselves induce SR-E1 expression, potentially creating a self-perpetuating inflammatory loop (108–110). Perturbing this feedback loop is a possible option to reduce inflammation-driven CVD in SLE (108, 111, 112).

#### 2.2.2 Calor: prostaglandins & thermoregulatory neurons

Fever can also be triggered by non-immune cells. Pro-inflammatory cytokines such as IL-1β, IL-6, and TNF-α stimulate cyclooxygenase-2 (COX-2), which catalyzes the production of prostaglandin E2 (PGE2). PGE2, in turn, binds to PGE2 receptors stimulating the part of the brain where thermoregulatory neurons modulate body temperature and induce fever (113). Elevated PGE2 has been detected in the cerebrospinal fluid (CSF) of patients with neuropsychiatric SLE (NPSLE), along with increased levels of IL-6, IgG, and autoantibodies against calf thymus antigens (114). Among these, CSF IL-6 has shown the strongest correlation with NPSLE severity and may serve as a potential biomarker (115, 116). In pristane-induced lupus mouse models, PGE2 mediates the production of proinflammatory cytokines, such as IL-6, IL-10, and IFN-*γ*, and NO (117). While no direct link between PGE2 and SRs in SLE has yet been confirmed, emerging evidence suggests functional intersections. For example, SR-B2–mediated microglial phagocytosis of amyloid-β is regulated by PGE2 receptor signaling in *in vivo* Alzheimer’s disease models (118). Additionally, celecoxib, a selective COX-2 inhibitor used in treating inflammation in SLE, has been shown to upregulate SR-B2 and downregulate SR-E1 in macrophages—indicating that prostaglandin pathways can modulate SR expression and potentially influence inflammatory outcomes (119).

#### 2.2.3 Calor: heat shock proteins

Heat shock proteins (HSPs), molecular chaperones released from cells undergoing stress, help maintain protein homeostasis (120). Incubating murine skin explants at fever-range hyperthermia (40°C) upregulates HSP70 expression and leads to dendritic cell (DC) migration (121). At similar temperatures, expression of HSP70 is induced in lymphocytes (122, 123). HSPs can help immune cells withstand and react to inflammatory environments (121).

HSPs are typically expressed intracellularly; however, under conditions of cellular stress (e.g., cell damage, necrosis), HSPs may be released extracellularly, where they act as DAMPs. Extracellular HSPs can prime antigen-presenting cells, promote cytokine production (e.g., TNF-α, IL-6), and contribute to the generation of autoantibodies (124–132). This immune activation correlates with HSP levels: more cell damage triggers higher HSP expression and a more intense immune response (133–135). Elevated HSP70 levels, HSP gene polymorphisms, and anti-HSP autoantibodies have all been associated with SLE pathogenesis (136–141).

Several SRs have been identified as HSP-binding receptors, including SR-E1, SR-F1, SR-A, SR-H1, and SR-L1 (141–145). SR-E1 and SR-F1 bind strongly to HSP70, HSP90, Grp94, Hsp110, and Grp170 (146–149). SR-B2 deficiency in LDL receptor–deficient mice exposed to hyperthermic stress leads to HSP70 overexpression and enhanced atherosclerosis, highlighting the interplay between SRs, heat shock responses, and vascular inflammation (150–152). In lupus-prone mice, HSP70-based DNA vaccines have shown promise in suppressing anti-dsDNA antibody production, reducing proinflammatory responses, promoting tolerogenic immune responses, and prolonging survival (153). Other therapeutic approaches, such as epitope-based immunization with HSP70-derived peptides, have demonstrated T_Reg_ activation without inducing systemic immunosuppression (154). SLE is associated with increased auto-antibody productions of other HSP-like proteins and HSPs, namely grp94 and calreticulin (155, 156), which has been found to play a role in during physiological stresses like fever (157) and in the pathogenesis of SLE (144, 158). SR-L1, an HSP receptor, is particularly relevant in this context. Membrane-bound SR-L1 mediates antigen presentation, cytokine secretion, and T helper cell priming (126, 159). SR-L1 plays an immunoprotective role by suppressing the expression of inflammatory mediators (MCP-1/CCL2, TNF-α, and MMP-9) (160, 161). To suppress inflammation, SR-L1 sheds its ectodomain, generating soluble SR-L1 (162), which can be detected in the serum of patients with SLE and Rheumatoid Arthritis (RA) (163). Bruton’s tyrosine kinase (Btk) also plays a key role by phosphorylating calreticulin on apoptotic cells (ACs), enabling membrane-bound SR-L1 to mediate the clearance of C1q-opsonized ACs. In the absence of Btk, SR-L1 cannot recognize calreticulin, resulting in the accumulation of apoptotic cell debris (164). Together, these findings suggest a multifaceted role for SRs in regulating immune responses to thermal and inflammatory stress. By binding HSPs and modulating cytokine production, SRs represent promising therapeutic targets and potential biomarkers for disease severity in SLE. Similarly, many HSPs themselves have been identified as targets for the treatment of autoimmunity, such as in RA, diabetes, multiple sclerosis (MS), and SLE (165, 166).

### 2.3 Dolor

In inflammation, *dolor* represents pain due to changes associated with perivasculature and nerve endings. Pain, especially chronic pain, is a hallmark of SLE and is often one of the first reported symptoms (167). Data shows that 85% of SLE patients report joint pain (168, 169) and 32%–66% report headaches (169). Up to 80% of SLE patients experiencing pain, fatigue, or joint pain rate these symptoms as moderate or severe (170). Pain in SLE can be both inflammatory and non-inflammatory in nature including musculoskeletal [arthritis, myositis, avascular necrosis, fracture, osteoarthritis (OA)], fibromyalgia), neuropsychological (headache, small fiber neuropathy), serositis (pericarditis, pleuritis, peritonitis), immunological disturbance, drug side effects, etc. as reviewed by Pisetsky et al. (171).

#### 2.3.1 Dolor: neuropathic pain

SR-L1 (known as LRP-1 or CD91), plays a major role in neuroinflammation, nerve de- and re-generation, and neuropathic pain (172, 173). SR-L1 deficiency in Schwann cells is linked to mechanical allodynia—pain from normally non-painful stimuli such as light touch—and impaired motor function, both of which contribute to peripheral nerve injury and chronic pain (174, 175). SR-L1 deletion on macrophages induces an increase in NFκB pathway activation and inflammation (176). SR-L1 downregulates proinflammatory cytokines (IL-1β, TNF-α, and IL-6) released due to NFκB activation. Perturbed SR-L1 across several cell types results in increased secretion (172).

Similarly, SR-L1 has a role in neuronal cell survival by modulating c-Jun N-terminal kinase (JNK)-mediated apoptosis (172). Perturbing SR-L1 leads to JNK pathway activation and the release of several proinflammatory cytokines (e.g., TNF-α and IL-1ß) and chemokines such as chemokine (C-C motif) ligand (CCL) 2 (CCL2), CCL3, or CCL4 (177–179). Consequently, SR-L1 downregulation promotes synaptic and neuronal loss, leading to cognitive impairment (172, 180–184). In MS, autoantibodies against SR-L1 have been found to inhibit function and contribute to poor clinical outcomes (185). In response to inflammatory triggers, membrane-bound SR-L1 has been found to be anti-inflammatory (178, 186–189); however, increased levels of soluble SR-L1 correlate with inflammation in patients with SLE and RA (163). SR-L1, through its ligands and its biologically active soluble form, has been explored as a potential therapeutic target for neuropathic pain, based on studies of axonal injury and Alzheimer’s disease (172, 177, 183). Although auto-antibodies to SR-L1 have not been attributed to SLE, auto-antibodies to scavenger receptor class L member 2 (SR-L2) have been found to be a major player in systemic autoimmune diseases (190). In a cohort of 147 patients, anti-LRP2 autoantibodies were detected in 87% with RA, 40% with SLE, 35% with systemic sclerosis, 15% with osteoarthritis, and 3% with Behçet’s disease (190).

Beyond their role in systemic autoimmunity, immune cells such as macrophages are increasingly recognized for their involvement in the pathophysiology of chronic pain (191–193). CD68 (SR-D1) is commonly used as a biomarker to quantify inflammatory cell and macrophage infiltration (194). Often associated with RA (195), Morton’s neuroma is an entrapment neuropathy characterized by compression of a plantar digital nerve in the foot, leading to neuropathic symptoms such as burning, paroxysmal pain, and paresthesia. In nerve samples from patients with Morton’s neuroma, increased intraneural CD68^+^ macrophages have been positively correlated with burning pain, while higher expression of SR-A6 has been linked to paroxysmal pain, as measured by the Neuropathic Pain Symptom Inventory (196). Similarly, intervertebral disc tissue from patients with lower back pain shows elevated infiltration of TNF-α^+^ and CD68^+^ cells (197). Conversely, in post-amputation patients experiencing phantom pain, nerve biopsies revealed a lower presence of CD68^+^ macrophages. This finding led researchers to propose a potential protective role for these cells against the development of chronic pain in certain contexts (191). These observations suggest that the role of infiltrating CD68^+^ macrophages in pain may vary depending on the condition and anatomical site. For example, pain in chronic pancreatitis—whether alcoholic, biliary, hereditary, or autoimmune in origin—is typically classified as inflammatory rather than neuropathic, though these categories are not mutually exclusive. Notably, in cases of chronic pancreatitis, CD68^+^ macrophage levels did not correlate with pain severity (194).

#### 2.3.2 Dolor: inflammatory pain

Building on the role of macrophage infiltration in various pain states, joint inflammation in diseases such as OA, RA, and SLE further illustrates the link between immune cell activity and pain. In these conditions, increased infiltration of immune cells and the production of pro-inflammatory mediators (often triggered by IgG immune complexes) sensitize and activate sensory nociceptors innervating joint tissues (198–201). Several SRs associated with macrophage activation have been implicated in inflammatory joint pain. For instance, elevated expression of CD163 or SF-I1 and the pro-inflammatory cytokine TNF-α correlates with higher resting pain scores in patients with hip OA (202). Similarly, IL-1β, another key pro-inflammatory cytokine, contributes to pain through its role in prostaglandin production (201, 203).

In RA murine models, macrophages expressing SR-D1, SR-I1, and SR-E3 are enriched in inflamed joints; however, a highly pathogenic and pro-inflammatory subset of macrophages co-expressing SR-I1 and SR-E3 has been identified in RA synovial tissue. These SR-E3^+^ SR-I1^+^ macrophages spontaneously secrete pro-inflammatory mediators including IL-6, IL-8, IL-1β, and TNF-α, and exhibit strong co-expression of CD40, a costimulatory activation marker known to drive chronic inflammation in RA (204–206). Remarkably, inhibition of CD40-TRAF6 signaling reversed the secretion of these mediators, suggesting that targeting this pathway may offer therapeutic benefit (207). Even more compelling is the observation that these pathogenic macrophages were present before the clinical onset of RA symptoms (207).

Extending beyond the joints, macrophage subsets expressing SR-E3 and SR-I1 have also been implicated in chronic pain development in the dorsal root ganglia (DRG). In mouse models genetically predisposed to chronic pain following peripheral injury, DRG-resident macrophages expressing SR-I1 alone (SR-E3^−^ SR-I1^+^) or both SR-E3 and SR-I1 (SR-E3^+^ SR-I1^+^) were found to promote chronic pain. Targeted depletion of these macrophage subsets effectively prevented the development of injury-induced chronic pain, highlighting their critical role in pain pathogenesis (208).

Relatively few SRs have been directly linked to pain in SLE or to pain mechanisms more broadly. However, further investigation into how SRs contribute to immune response pathways in SLE, particularly those leading to neuropathic and inflammatory pain, may reveal additional SRs involved in early immune dysregulation and the onset of autoimmunity. Uncovering these connections could offer novel insights into both SLE pathogenesis and pain modulation.

### 2.4 Tumor

The classical hallmark of *tumor* in inflammation, or swelling, is primarily caused by increased vascular permeability, leukocyte infiltration, and fluid accumulation in affected tissues (22). A key event in this process is diapedesis, or trans-endothelial migration, in which leukocytes exit the bloodstream and traverse the vascular and lymphatic endothelial barriers to reach sites of inflammation (209). This involves a tightly regulated sequence: leukocyte recruitment, adhesion to ECs, and eventual transmigration into surrounding tissues (210). Persistent vascular inflammation is a hallmark of several autoimmune conditions, including SLE (211, 212), where dysregulated immune cell infiltration exacerbates tissue injury and chronic inflammation.

#### 2.4.1 Tumor: leukocyte recruitment & transmigration

Effective leukocyte recruitment is essential for host defense and tissue repair; however, in autoimmune diseases such as SLE, excessive or uncontrolled leukocyte accumulation leads to chronic inflammation and tissue damage (210).

Myeloid cells, particularly neutrophils and monocytes, play a central role in initiating and sustaining vascular inflammation in SLE (212–215). Neutrophils contribute to inflammation not only through phagocytosis but also by producing reactive oxygen species (ROS), releasing neutrophil extracellular traps (NETs), and modulating adaptive immunity via crosstalk with dendritic cells, macrophages, and lymphocytes (211, 216, 217). SRs, particularly SR-A, have been implicated in neutrophil activation, including via mitogen-activated protein kinase (MAPK) signaling pathways, leading to increased production of proinflammatory cytokines (i.e., IL-6, TNF-α) and NET formation (218).

SLE is also characterized by elevated Type I interferon levels, which drive monocyte chemotaxis through increased expression of MCP-1 and MIP-1*α*. This promotes the upregulation of SRs such as SR-A and SR-B2, further enhancing monocyte and neutrophil activation (219–223). These receptors facilitate the uptake of modified low-density lipoproteins (LDLs), linking innate immune activation with lipid metabolism and vascular inflammation. For example, SR-B2 supports macrophage spreading and migration (224), inflammasome activation (225), and has been implicated in lesional macrophage proliferation (223, 226).

#### 2.4.2 Tumor: diapedesis & SR-mediated endothelial crosstalk

The transmigration of leukocytes across the endothelium is a tightly regulated, multi-step process involving changes in both leukocytes and endothelial cells. Inflammatory cytokines upregulate endothelial adhesion molecules, while chemokines activate leukocyte integrins to promote firm adhesion and extravasation (210). Leukocytes then alter their morphology to traverse the endothelial barrier and surrounding pericyte layer, ultimately infiltrating the inflamed interstitial tissue (210).

Among scavenger receptors, SR-G1 is unique in exhibiting both receptor and chemokine-like functions. In its membrane-bound form, SR-G1 binds oxLDLs and phosphatidylserine (PS), contributing to phagocytosis and waste clearance. In its soluble form, SR-G1 acts as a chemoattractant through its interaction with the CXCR6 receptor on bone marrow plasma cells and T cells (227). Soluble SR-G1 is particularly relevant to SLE: elevated serum levels correlate with disease severity, organ involvement, and prognosis in both adult (228) and juvenile SLE (229). Levels decrease with effective treatment, supporting its potential utility as a biomarker for SLE progression and therapeutic response (228).

#### 2.4.3 Tumor: SRs in endothelial dysfunction

A major complication in SLE is the premature development of atherosclerotic cardiovascular disease (ASCVD). Early plaque formation is marked by endothelial dysfunction and the infiltration of pro-inflammatory leukocytes beneath the endothelial monolayer (226, 230). Monocyte-derived macrophages proliferate in response to hematopoietic growth factors such as macrophage colony-stimulating factor (M-CSF) and accumulate cholesteryl esters, forming foam cells (231–234). M-CSF also enhances SRs expression, particularly SR-A and SR-B2, promoting oxLDL uptake and foam cell formation (233, 235).

SR-B2 plays a central role in this process. Suppression of SR-B2 in murine models reduces aortic lesion size, suggesting that its function is non-redundant and critical in lesion development (236, 237). Importantly, SLE patient blood samples contain higher levels of oxLDL-containing immune complexes (238), which upregulate SR-B2 expression in healthy cells exposed to this SLE plasma (235). This mechanism likely contributes to accelerated foam cell formation and atherosclerosis in SLE, positioning SR-B2 as a potential therapeutic target (239, 240).

Additionally, Mer tyrosine kinase (MerTK), another SR, is essential for the clearance of apoptotic cells within plaques. Loss of MerTK impairs efferocytosis, promoting the formation of a lipid-rich necrotic core and driving plaque instability (241) as well as T cell–mediated β cell autoimmunity (242). Elevated levels of soluble MerTK in SLE patients correlate with disease activity (243), complement depletion, and anti-dsDNA titers, suggesting a role in both cardiovascular and autoimmune pathology (243, 244).

Another key player is SR-E1 (LOX-1), which mediates uptake of modified LDLs and contributes to foam cell formation. OxLDL exposure reduces DNA methylation of the SR-E1 promoter, creating a positive feedback loop that amplifies SR-E1 expression and promotes plaque progression (245). In SLE patients with ASCVD, serum levels of soluble SR-E1 correlate with inflammatory biomarkers such as high-sensitivity C-reactive protein (hsCRP), proinflammatory HDL, and oxLDL (108). Furthermore, higher sSR-E1 levels are associated with earlier age of SLE diagnosis (108), suggesting that SR-E1 could serve both as a biomarker and a therapeutic target in SLE-related vascular disease.

#### 2.4.4 Tumor: endothelial cell scavenger receptors and tissue crosstalk

In SLE, the endothelium has been shown to be in a dysregulated state, even during low disease activity (246). SRs are expressed on endothelial cells, where they regulate vascular permeability, immune cell trafficking, and antigen presentation. These scavenger endothelial cells (SECs) are especially prominent in the liver, where hepatic sinusoidal endothelial cells (HSECs) act as filters for bloodborne antigens and macromolecules, given the liver’s extensive exposure to gut-derived microbial products (247–251). HSECs express a range of SRs including SR-H1 (252–254), SR-H2 (255), SR-B2 (256), SR-B1 (257), SR-E3 (258, 259), and SR-F1 (250). SR-H1, for example, is induced by proinflammatory stimuli (260) and, *in vitro*, regulates lymphocyte trafficking to inflamed tissues (261). SR-H1 expression on monocytes is considered a predictive biomarker for increased cardiovascular-related disease risk (13). Both SR-H1 and SR-H2 bind to a variety of ligands including acLDL, advanced glycation end-products (AGEs), and both gram-positive and gram-negative bacteria (13, 262). SR-H1 also facilitates T and B cell trans-endothelial migration via interactions with adhesion molecules like ICAM-1 and VAP-1 (13, 253, 263–266).

Interestingly, SR-H1 promotes antigen presentation in a tolerogenic context by cross-presenting exogenous antigens on MHC-I and MHC-II molecules with high expression of inhibitory ligands (e.g., PD-L1), HSECs help induce regulatory T cells rather than proinflammatory responses (267–271). Inhibition of SR-H1 and SR-H2 has been shown to induce an anti-inflammatory plasma proteome and reduce monocyte-driven atherogenesis, pointing to their therapeutic potential (272).

SR-F1 (SCARF1, SREC-1), initially identified in human umbilical vein endothelial cells (264), is also upregulated in chronic liver diseases such as primary sclerosing cholangitis (PSC), primary biliary cholangitis (PBC), ALD, and non-alcoholic steatohepatitis (NASH) (250). SR-F1 activation by TNF-α or LPS enhances CD4+ T cell recruitment, working in concert with VCAM-1 to facilitate immune cell adhesion during liver inflammation (250). Other SRs, including SR-E1 (273, 274), SR-H1 (253, 254, 275), SR-H2 (255) also mediate leukocyte adhesion to endothelial cells, contributing to inflammatory crosstalk between the vasculature and the immune system.

#### 2.4.5 Tumor: SRs in regulating neutrophil NETS and endothelial injury

Neutrophils recruited to the endothelium are activated by immune complexes (276) and perform several effector functions including phagocytosis, degranulation, and formation of neutrophil extracellular traps (NETs) (276, 277). While NETs are antimicrobial, excessive NET formation (NETosis) and elevated levels of circulating NETs is pathogenic in SLE, where it contributes to endothelial injury, immune complex formation, and the development of autoantibodies (277).

Increased NETs have been observed in patients with LN and in MRL-*lpr* mice (278). SR-J1 (RAGE) is an SR expressed on endothelial cells that plays a role in triggering NETosis (279) and, together with clathrin, mediates the uptake of NETs. However, endothelial phagocytic capacity is limited, and NET overload disrupts vascular integrity. Specifically, NET-associated elastase degrades VE-cadherin at intercellular junctions, increasing vascular permeability and leakage (278). This mechanism links NETosis and SR-mediated uptake to vascular damage and albumin extravasation, ultimately highlighting the role of NET clearance in preserving endothelial barrier function in SLE.

### 2.5 Functio laesa

*Functio laesa*, Latin for “loss of function,” can refer to either impaired organ function (280) or neurological responses to inflammation and pain (281). In the context of systemic inflammation, such as in SLE, *functio laesa* often reflects multi-organ dysfunction or outright failure (282). SLE can affect nearly every organ system, manifesting in complications such as lupus nephritis (kidneys), neuropsychiatric disease (central nervous system), cutaneous lupus (skin), lymphadenopathy (lymphatic system), and various cardiovascular conditions (283, 284). Organ damage occurs in at least 50% of SLE patients (285), though some studies report even higher rates. A Taiwanese cohort found that over 80% of SLE patients developed organ damage within 6 months of diagnosis (286), while U.S. studies report damage in 33%–50% of patients within the first five years (287). The most frequently affected systems vary by region: ocular, neuropsychiatric, and cardiovascular systems are common in the U.S. and Germany (287, 288), while renal, neuropsychiatric, pulmonary, gastrointestinal, and cutaneous systems are prominent in Taiwanese patients (286, 289).

#### 2.5.1 Functio laesa: neuropsychiatric

Neuropsychiatric symptoms are among the most common manifestations of SLE, affecting 80%–90% of patients worldwide (290). These symptoms include cognitive impairment, motor dysfunction, sleep disruption, fatigue, mood disorders, and behavioral changes. Cognitive dysfunction often presents early—prior to the development of dementia or confusion—whereas symptoms like depression or headaches are more difficult to diagnose and frequently missed in early screenings (290).

Up to 40% of neuropsychiatric SLE (NP-SLE) symptoms arise before or at the time of SLE diagnosis, and 60% typically develop within a year (291, 292). Current research is focused on whether NP symptoms are driven by central nervous system (CNS) inflammation in chronic SLE, CNS dysfunction and damage, or treatments and medications (290, 293). While lesions are not always evident in NP-SLE patients, functional abnormalities have been identified, including cerebral hypoperfusion (292, 294–298), metabolic deficiencies (292, 299–302), and—most commonly—progressive neuronal atrophy (292, 298, 300, 303–310).

In SLE-prone mouse models, neuropsychiatric and behavioral symptoms precede systemic autoimmunity, immune cell infiltration, or vascular damage (311). Interestingly, microglia in SLE-prone mice exhibit neurodegenerative disease-associated signatures prior to systemic SLE manifestations. Microglia from SLE mouse models show upregulation of genes involved in SR activity and downregulation of genes involved in inflammation and chemotaxis, suggesting that microglia in SLE-prone mice may not be able to regulate inflammation appropriately (311). Makinde *et al*. found SRs, SR-G1 (CXCL16) and LGALS3BP, are upregulated in lupus-prone mouse microglia. In healthy conditions, microglial cells maintain tissue homeostasis in the brain by sensing changes in the environment and responding appropriately. Anti-inflammatory microglia release immunomodulatory factors to support tissue repair in the brain. In disease contexts, anti-inflammatory microglia may also reduce immune response and promote cell invasion and tumorigenesis (312). Outside of SLE, microglial SR-G1 has been proposed as a therapeutic target to reduce neuroinflammation (312), and LGALS3BP may be a therapeutic target for its roles in angiogenesis and tumor progression (313). In SLE, serum SR-G1 and platelet LGALS3BP levels also correlate with LN severity (229, 314), pointing to systemic involvement beyond the CNS.

#### 2.5.2 Functio laesa: tissue homeostasis & clearance of apoptotic cells

Dysregulation of both innate and adaptive immune responses contribute to SLE (315). Adaptive immunity establishes a highly specific immunological memory to pathogens. B and T cells of the adaptive immune system are responsible for ensuring a specific, controlled spatiotemporal response to limit or prevent excessive tissue damage (316). The breakdown in immune tolerance is a hallmark of SLE development (315), as well as many other autoimmune disorders (316). Tissue homeostasis depends on the efficient clearance of apoptotic cells (ACs) by phagocytes in an immunologically silent process that prevents inflammation (317). Dying cells release “find-me” signals that attract phagocytes, while surface markers like phosphatidylserine (PS) serve as “eat-me” signals (317). Failure to clear ACs leads to their secondary necrosis, triggering inflammation, disruption of self-tolerance, and immune activation—a major contributor to SLE pathogenesis (318–321).

Elevated levels of uncleared ACs in SLE patients support the notion that defective clearance contributes to disease (319, 320). Scavenger receptors are critical in mediating efferocytosis, including SR-A, SR-F, and SR-H families (11, 262, 264, 322–325). SR-A1 and MARCO (SR-A6) are key receptors in this process. SR-A1 expressed on thymic macrophages was first shown to mediate apoptotic thymocyte clearance, and its blockade reduces phagocytosis by ~50% (326). Mice lacking SR-A1 and MARCO develop higher levels of autoantibodies and lupus-like disease due to impaired clearance of apoptotic debris by marginal zone macrophages in the spleen (323). Additionally, SLE patients and murine models have been found to spontaneously produce autoantibodies against these SRs, impairing the apoptotic cell removal ability of these two SRs (323, 327).

SR-F1 (SCARF1) also plays a role in apoptotic cell clearance. Mice lacking SCARF1 develop lupus-like symptoms, including nephritis and dermatitis, driven by defective phagocytosis of PS- and C1q-labeled apoptotic cells (328). SCARF1 is a non-redundant efferocytosis receptor expressed on BDCA1^+^ dendritic cells, where its engagement promotes anti-inflammatory IL-10 production via STAT1/STAT3 signaling (329). Interestingly, while SCARF1 expression is not reduced in SLE patients, they exhibit anti-SCARF1 autoantibodies, which correlate with impaired efferocytosis (329). Additional work is needed to understand the function and action of the anti-SCARF1 autoantibodies, and whether these autoantibodies can be used as biomarkers for SLE.

#### 2.5.3 Functio laesa: systemic organ damage

SLE-associated systemic inflammation leads to damage in organs such as the heart, lungs, and liver, particularly in patients with hematological symptoms like leukopenia or thrombocytopenia due to the circulation and accumulation of auto-antibodies and inflammatory mediators throughout the body (330). Scavenger receptors are again implicated—SR-A expression on peripheral blood mononuclear cells (PBMCs) correlates with systemic inflammatory response syndrome (SIRS) and multiple organ dysfunction syndrome (MODS), both indicators of poor survival outcomes (331). Further, mechanistic studies further demonstrate that MSR1 (SR-A) can physically synergize with Toll-like receptor 4 to amplify NF-kB signaling and pro-inflammatory cytokine release, providing a potential molecular bridge to the cytokine storm characteristic of MAS leading to systemic organ damage (332, 333). Similarly, soluble SR-I1 (CD163) levels correlate with MODS severity and prognosis in sepsis (334). Increased SR-I1 expression is found on macrophages in Macrophage Activation Syndrome patients who later develop SLE (65) and on PBMCs of prior diagnosed SLE patients (335). In LN and glomerulonephritis (GN), CD163^+^ macrophage infiltration is positively associated with disease severity and renal function decline (336, 337).

Effective SR-mediated clearance of dying cells is protective in acute injury, limiting the release of intracellular contents and preventing secondary necrosis and inflammation (338, 339). Conversely, impaired clearance promotes chronic inflammation and autoimmunity, as seen in SLE (340). The soluble scavenger receptor CD5l (also known as AIM, apoptosis inhibitor of macrophage) binds cellular debris and promotes phagocytic internalization of dead cells. AIM is primarily expressed by macrophages in the liver, lymphoid, and inflamed tissues (338, 341, 342). Elevated AIM levels are observed in both SLE patients (343) and lupus prone mice (344), correlating with SLE disease activity (343). AIM is most well-known for its role in Acute Kidney Injury (AKI) and chronic liver injury in which the accumulation of circulating AIM correlates with the progression of organ damage (345, 346). Two other SRs, SR-B2 and kidney injury molecule-1 (KIM-1), can recognize AIM when bound to cellular debris (338, 347). KIM-1, a scavenger receptor not typically expressed in healthy kidneys, is markedly elevated in injured renal tissue (348, 349). Elevated urinary KIM-1 levels in SLE patients suggest its utility as a non-invasive biomarker for renal involvement and progression of LN (350).

### 2.6 Fluor

Fluor refers to the secretion of mucus and inflammation of mucous membranes (19). Patients with autoimmune diseases, including SLE, exhibit an increased risk of developing chronic sinusitis (351, 352). Interestingly, chronic sinusitis may also serve as an early indicator of autoimmune disease onset (353). This relationship is thought to arise either from intrinsic immune dysregulation—leading to impaired mucosal defense and tolerance breakdown—or from extrinsic factors such as medications or infections that trigger or exacerbate autoimmunity (354).

One key player in mucosal immunity is glycoprotein-340 (gp-340), a member of the SR family (355, 356). Gp-340 is highly expressed in the sinonasal, ocular, and pulmonary mucosa, where it contributes to innate defense through pathogen recognition and clearance (355–357) and is found to be up-regulated in patients with chronic sinusitis (357).

Gp-340 functions in part through its interaction with Surfactant Protein D (SP-D), a collectin involved in pathogen recognition, modulation of inflammation, and phagocytosis (355, 358, 359). In SP-D-deficient mice, bacterial lung infections result in elevated inflammation, increased oxidative stress, and impaired macrophage function (360). Low circulating levels of SP-D have also been linked to the development of SLE (359). Although a direct mechanistic connection between gp-340 and SLE has not yet been established, its encoding gene, Deleted in Malignant Brain Tumor 1 (DMBT1), has been associated with several autoimmune and immune-related conditions, including SLE (361, 362).

A similar compromise in mucosal barrier integrity is seen in the gastrointestinal tract, which plays a vital role in immune homeostasis. The intestinal epithelium and its associated mucosal layer protect against microbial invasion while allowing for nutrient absorption (363, 364). Increased intestinal permeability, commonly referred to as “leaky gut syndrome”, permits the translocation of microbial products into the bloodstream, triggering both acute and systemic inflammation (363, 364). “Leaky Gut Syndrome” is increasingly implicated in the pathogenesis of SLE, as it has been in other autoimmune diseases such as RA, multiple sclerosis (MS), and type 1 diabetes (365).

In lupus, impaired gut barrier function allows pathogen-associated molecular patterns (PAMPs) and DAMPs to enter the bloodstream, activating dendritic cells, macrophages, and neutrophils (364). Bacterial components such as lipopolysaccharide (LPS), lipoteichoic acid (LTA), and β-glucans have been detected in the serum of SLE patients, reflecting microbial translocation and systemic immune activation (366). Scavenger receptor SR-E3 (CD206), also known as the mannose receptor, has been implicated in both gut dysbiosis and LN (367). In lupus-prone mouse models, colonization with *Segmented Filamentous Bacteria* (SFB) exacerbates glomerulonephritis, alters immune cell profiles, and disrupts gut barrier integrity (368). Notably, kidney-infiltrating CD206^+^ macrophages were observed in SFB-colonized mice, suggesting that gut-primed immune cells migrate to distal inflammatory sites, such as the kidney (368). In human SLE patients, the presence of CD206^+^ macrophages in the kidney correlates with disease severity (369).

The importance of mucosal SRs in maintaining immune tolerance is further supported by findings in Celiac disease, another autoimmune disorder affecting the gastrointestinal mucosa. Like SLE, Celiac disease is characterized by impaired clearance of ACs, which leads to the accumulation of cellular debris and chronic inflammation. Studies have shown that duodenal tissues from Celiac patients exhibit reduced expression of several scavenger receptors, including SR-B2, thrombospondin-1 (TSP-1), and CD61, alongside elevated levels of inflammatory cytokines such as IL-15, IL21, and IFN-γ (370). Furthermore, lamina propria mononuclear cells (LPMCs) isolated from Celiac patients display a diminished capacity to phagocytose ACs (370). Disruption in these localized immune defenses may initiate or amplify systemic inflammation and contribute to disease pathogenesis.

## 3 Scavenger receptors as biomarkers and targets to treat SLE

The complexity and heterogeneity of SLE—both in terms of clinical presentation and underlying immunological mechanisms—have long posed significant challenges in diagnosis, prognosis, and treatment. While serological markers such as anti-dsDNA and anti-Smith antibodies are commonly used, they lack sensitivity and specificity across all disease stages and patient populations. Therefore, there is growing interest in identifying novel, more reliable biomarkers that reflect disease activity, organ involvement, and therapeutic responsiveness.

Among the most promising candidates are SRs. SRs play a critical role in innate immunity through the recognition and clearance of endogenous and exogenous ligands, including apoptotic cells, modified lipids, and microbial products. Dysregulated expression or function of SRs has been implicated in the breakdown of self-tolerance and chronic inflammation, hallmarks of SLE pathogenesis. Notably, SRs also participate in intracellular signaling cascades such as the MAPK, NF-κB, and JAK-STAT pathways—many of which are known to be dysregulated in autoimmune conditions (316, 371–373). This positions SRs not only as mediators of immune response but also as potential biomarkers for disease progression and targets for immunomodulatory therapy.

Biomarkers are essential for the diagnosis, prognosis and the monitoring of SLE. Markers like dsDNA, complement levels and the presence of certain autoantibodies have been validated and are being used in clinical practice. However, it wasn’t until 2007 that Wermeling et al. showed the presence of antibodies responsible for recognizing MARCO and SR-A (323). The discovery was then followed up by Chen et al., where the group identified an increase of anti-SR-A and anti-MARCO IgG in SLE patients when compared to controls (327, 374). In 2022, Jorge et al. showed an increase in anti-SCARF1 antibodies that correlate with increase dsDNA in the serum (329). This data was true for 26% of the patients suffering from SLE, however no additional correlation was found with other disease markers. Recently it was identified that the SR sCD163 (SR-I1) was elevated in patients suffering from LN, and it was suggested to be used as a prognostic biomarker (59). These discoveries are only in the initial stages and additional work is necessary to take this finding to the clinic. There is one thing all these discoveries had in common, the presence of these antibodies to SRs contribute to the breakdown of self-tolerance and increase autoimmune pathogenesis, making SRs the perfect candidate as disease progression biomarker.

Current SLE management strategies aim to (1) minimize disease activity, (2) prevent irreversible organ damage, (3) reduce the burden of comorbidities and treatment-related side effects, and (4) improve quality of life by alleviating pain and fatigue (5). However, due to the heterogeneous nature of the disease, a universal diagnostic or treatment protocol remains elusive. While glucocorticosteroids and antimalarial drugs have historically formed the backbone of treatment, their long-term use is associated with serious side effects and complications (Table 2). In recent years, the therapeutic landscape has shifted toward biologics, with only two FDA-approved options currently available—belimumab and anifrolumab (1, 375–377). Despite their promise, many patients exhibit incomplete or variable responses, necessitating adjunct or personalized therapies that account for unique immune signatures and organ involvement (375).

Targeting key signaling pathways associated with SR activation—such as MAPK, NF-κB, and JAK-STAT—has been explored in clinical trials with mixed outcomes, largely due to SLE’s intrinsic heterogeneity and overlapping immunopathogenic mechanisms (101, 316, 372, 373). These challenges underscore the need for refined biomarkers and therapeutic targets. As research advances, scavenger receptors may provide dual utility as both indicators of disease activity and modulators of inflammation, offering a promising avenue for stratified medicine in SLE care.

## 4 Discussion

Scavenger receptors play an essential role in the immune system and their immunomodulatory function through a variety of immune and non-immune cell types in the context of inflammation and several autoimmune diseases. The past two decades of research have shown that the role of SRs in inflammatory disease might have been underestimated, and the more recent body of published work demonstrates the many ways SRs influence, modulate, and directly contribute to inflammatory disease-related pathways.

Although great strides have been made to understand how SRs contribute to inflammation, many unanswered questions remain. As shown in this review, there is quite a bit of redundancy regarding SRs and the pathways that they participate in or regulate. Still, it appears that one class is not responsible for the immunomodulatory effects of SRs (10, 260). SRs participate in pro- and anti-inflammatory signaling cascades, which muddies their contribution to the development and prognosis of inflammatory disease as well. Furthermore, they interact with other pattern recognition receptors to enhance or suppress a response (10, 378).

There is convincing evidence that some SRs can act as potentially useful biomarkers for autoimmune disease diagnostics and prognostics, but this is not the case for every SR family. For example, autoantibodies for SR-AI are elevated in SLE patients (327) and autoantibodies to SCARF1 are linked to defects in efferocytosis and autoimmunity (329). Further research is necessary to identify SR with subset or symptoms of autoimmunity. However, designing SR-based therapeutic approaches may prove challenging since SR levels do not necessarily vary between healthy individuals and those with inflammatory disease. Downstream research is needed to finetune current findings and provide greater clarity on these knowledge gaps.

Despite the knowledge gaps that remain regarding SRs and their role in inflammation and autoimmunity, current research demonstrates that SRs wear many immunological hats and contribute to a variety of inflammatory signaling pathways. Therefore, it is crucial to continue investigating the role of SRs in inflammation to determine the therapeutic potential of targeting SRs in the context of inflammatory and autoimmune disease.

## Supplementary Material

table

The Supplementary Material for this article can be found online at: https://www.frontiersin.org/articles/10.3389/flupu.2025.1679564/full#supplementary-material

## Figures and Tables

**TABLE 1:** Consensus nomenclature and common names of scavenger receptors.

Consensus Nomenclature	Common Names
SR-A1	MSR1, SR-AI, SCARA1
SR-A1.1	SR-AII
SR-A3	APC7, MSRL1, SCARA3
SR-A4	COLEC12, CL-P1, SRCL
SR-A5	SCARA5, TESR
SR-A6	MARCO
SR-B1	CD36L1, SR-BI
SR-B2	CD36, PAS4, SCARAB3
SR-D1	CD68
SR-E1	LOX-1, OLR1
SR-E2	Dectin-1, CLEC7A
SR-E3	CD206, Mannose Receptor 1, MRC1
SR-E4	CLEC4HI, HL-1
SR-F1	SCARF1, SREC-1
SR-F2	MEGF10
SR-G1	CXCL16, SR-PSOX
SR-H1	STAB1, FEEL-1
SR-H2	FEEL-2
SR-I1	CD163
SR-J1	RAGE
Unclassified/Noncanonical (SRCR)	CD5l (AIM)1, MerTK, gp-340, KIM1

Scavenger receptor cysteine rich (SRCR) is a superfamily of receptors that is characterized by the presence of a conserved ~100–110 cysteine rich structure. On this review we focus on AIM as the main SRCR.

**TABLE 2:** Current SLE treatment options.

Drug	Class	Molecular target	EULAR domains	SLE approval	Ref.
Azathioprine	Non-steroidal immunosuppressant	Calcineurin inhibitor	Hematologic, Skin, Renal, Constitutional, Neuropsychiatric	FDA, EMA	(375, 379, 380)
Cyclosporine	Non-steroidal immunosuppressant	Calcineurin inhibitor	Renal, Mucocutaneous, Hematologic	OL	(375, 381)
Cyclophosphamide	Non-steroidal immunosuppressant/ Cytotoxic	Add an “alkyl” group to DNA	Renal, Neuropsychiatric, Hematologic	OL	(375, 379, 380)
Mycophenolate mofetil	Non-steroidal immunosuppressant	Inosine Monophospate Dehydrogenase inhibitor	Hematologic, Mucocutaneous, Renal	OL	(375, 382)
Abatacept	Non-steroidal immunosuppressant	CD80/CD86 on APCs blocker	Hematologic, Renal, Mocucutaneous	OL	(379)
Voclosporin	Non-steroidal immunosuppressant	Calcineurin inhibitor	Renal, Mucocutaneous, Hematologic	FDA, EMA	(375, 380)
Tacrolimus	Non-steroidal immunosuppressant	Calcineurin inhibitor	Renal, Mucocutaneous, Hematologic	OL	(375, 380)
Methotrexate	Non-steroidal immunosuppressant/ Cytotoxic	Calcineurin inhibitor	Arthritis, Mucocutaneous, Serositis, Musculoskeletal	OL	(375, 380)
Belimumab	Biologic	BlyS neutralizing	Arthritis, Mucocutaneous, Renal, Musculoskeletal, Immunological	FDA, EMA	(375, 379, 380)
Anifrolumab	Biologic	Type I IFN Receptor blocker	Arthritis, Mucocutaneous,	OL	(375, 380)
Rituximab	Biologic	Binds to CD20	Neuropsychiatric, Renal, Arthritis	OL	(375, 380)
Chloroquine	Antimalarial	Inhibit autophagy	Hematologic	OL	(375)
Hydroxychloroquine	Antimalarial, Antirheumatic	IFN suppressor	Arthritis, Mucocutaneous, Serositis	FDA, EMA	(375, 379, 381)
Quinacrine	Antimalarial	Anti-inflammatory	Mucocutaneous, Serositis, Arthritis	FDA	(375, 380)
Methylprednisolone	Glucocorticoids	Anti-inflammatory	Hematologic, Renal, Neuropsychiatric	FDA	(375)
Dexamethasone	Glucocorticoids	Binding to the cytoplasmic glucocorticoid receptor (GR)	Renal, Neuropsychiatric	FDA	(375)
Prednisolone	Corticosteroids	Multiple	Overactive immune system	FDA, EMA	(379)
Hydrocortisone	Corticosteroids	Binding to the cytoplasmic glucocorticoid receptor (GR)	Overactive immune system	FDA, EMA	(379)
Aspirin	NSAID	Thrombosis prevention	Serositis, Antiphospholipid antibody, Arthritis, Constitutional	FDA	(379)
Celecoxib	NSAID	COX-2 inhibitor	Arthritis, Constitutional	OL	
Heparin	Anticoagulant	Thrombin inhibitor and clotting factors	Serositis, Antiphospholipid antibody,	FDA	(382)
Warfarin	Anticoagulant	Interferes with clotting factors	Serositis, Antiphospholipid antibody,	FDA	(382)

* OL: Off-label medications.
