# Supplementary material for: Scavenger receptors: key players in the immunological puzzle of lupus
Source: Front Lupus. Author manuscript; Available in PMC 2026 Feb 5. (PMC12872056; doi:10.3389/flupu.2025.1679564)
Supplement: table [file NIHMS2135362-supplement-table.docx]

|  | | | **Class A**  **(SR-A)** | | | | | | **Class B**  **(SR-B)** | | | **Class D**  **(SR-D)** | **Class E**  **(SR-E)** | | | | **Class F**  **(SR-F)** | | | **Class G (SR-G)** | **Class H**  **(SR-H)** | | **Class I (SR-I)** | | | **Class J**  **(SR-J)** | | **Class K**  **(SR-K)** | **Class L**  **(SR-L)** | | **Unclassified SRs or Proteins with SR functions** |
| --- | --- | --- | --- | --- | --- | --- | --- | --- | --- | --- | --- | --- | --- | --- | --- | --- | --- | --- | --- | --- | --- | --- | --- | --- | --- | --- | --- | --- | --- | --- | --- |
| **Members** | | | ***SR-A1*** | ***SR-A1.2*** | ***SR-A3*** | ***SR-A4*** | ***SR-A5*** | ***SR-A6*** | ***SR-B1*** | ***SR-B1.1*** | ***SR-B2*** | ***SR-D1*** | ***SR-E1*** | ***SR-E2*** | ***SR-E3*** | ***SR-E4*** | ***SR-F1*** | ***SR-F2*** | ***SR-F3*** | ***SR-G*** | ***SR-H1*** | ***SR-H2*** | ***SR-I1*** | ***SR-I2*** | ***SR-I3*** | ***SR-J1*** | ***SR-J1.1*** | ***SR-K1*** | ***SR-L1*** | ***SR-L2*** |  |
| **Aliases** | | | SCAR-A1, CD204, MSR1 [1, 2] | Splice variant of SR-A1, SR-A2 [1, 2] | SCAR-A3, MSRL1, APC7, CSR1 [1, 2] | COLEC12, SCAR-A4, SRCL, or CL-P1 [1, 2] | SCAR-A5 or TESR [1, 2] | SCAR-A2 or MARCO [3-6] [1, 2] | SCARB1, SR-BI CD36L1 [1, 2] | LIMP2, CD36L2, LGP85, SCARB2 [1, 2] | CD36, SCARB3, PAS4, FAT, GPIV [1, 2] | CD68, gp110, SCARD1, LAMP4 [1, 2] | LOX-1, OLR1, SCARE1, CLEC8A [1, 2] | Dectin-1, CLEC7A [1, 2] | Mannose receptor 1, CD206, MRC1 [3, 7] | Asialoglycoprotein receptor 1 (ASGPR1), CLEC4H1, HL-1 [3, 7] | SREC1, SCARF-1 [1, 2] | SREC2, SCARF-2 [1, 2] | MEGF10, SCARF-3, EMARDD [1, 2] | SR-PSOX, CXCL16 [1, 2] | FEEL-1, Stabilin-1, STAB1,CLEVER-1 [1, 2] | FEEL-2, Stabilin-2, STAB2, HARE [4] [1, 2] | SCART1, CD163, M130 [1, 2] | M160, CD163B, CD163L1 [1, 2] |  | RAGE or AGER [1, 2] | sRAGE, esRAGE [1, 2] | CD44 | CD91/LRP1/APOER | gp330/LRP2/megalin |  |
| **Localization & Expression** | **Endothelial Cells** | | X [8] [3, 7] | X [3, 7] | X [3, 7] | X [3, 7] | X [3, 7] | X [3, 7] | X [9] |  | X [10, 11] [12] |  | X [8] [13, 14] |  | X [15, 16] |  | X [8] [17] |  |  | X [8] | X [8] [18-20] | X [3, 7] [21] |  |  |  | X [8] [22] | X [8] |  |  |  |  |
|  | **Epithelial Cells** | |  |  | X [3, 7] | X [3, 7] | X [8] [3, 7] |  |  |  |  |  |  |  |  |  |  |  |  |  |  |  |  |  |  |  |  |  |  |  |  |
|  | **Neuronal Cells** | |  |  |  |  |  |  |  |  |  |  |  |  |  |  | X [8] |  |  |  |  |  |  |  |  |  |  |  |  |  |  |
|  | **Vascular Smooth Muscle Tissue** | | X [8] |  |  |  |  |  |  |  | X [10, 11] |  | X [8] [23] |  |  |  |  |  |  | X [8] |  |  |  |  |  |  |  |  |  |  |  |
|  | **Smooth Muscle Tissue** | |  |  |  |  |  |  |  |  |  |  |  |  |  |  |  |  |  |  |  |  |  |  |  | X [8] | X [8] |  |  |  |  |
|  | **Skeletal Muscle** | |  |  |  |  |  |  |  |  | X [10, 11] |  |  |  |  |  |  |  |  |  |  |  |  |  |  |  |  |  |  |  |  |
|  | **Kupffer Cells** | | X [3, 7] | X [3, 7] | X [3, 7] | X [3, 7] | X [3, 7] |  |  |  |  |  |  |  |  |  |  |  |  |  |  |  |  |  |  |  |  |  |  |  |  |
|  | **Microglia** | |  |  |  |  |  |  |  |  | X [10, 11] [24] |  |  |  |  |  |  |  |  | X [25] |  |  |  |  |  |  |  |  |  |  |  |
|  | **Macrophages** | | X [8] [6] [26] [3, 7] | X [3, 7] | X [3, 7] | X [3, 7] | X [3, 7] | X [8] [26] [3, 7] |  | X [8] | X [10, 11] [27] | X [8] [28] | X [27] | X [3, 7] | X [29] [30-32] |  | X [3, 7] |  |  | X [8] | X [8] | X [8] | X [8] [29] [30-32] | X [3, 7] |  | X [8] | X [8] |  | X [33, 34] [35] |  |  |
|  | **Dendritic Cells** | | X [6] [3, 7] | X [3, 7] | X [3, 7] | X [3, 7] | X [3, 7] | X [3, 7] |  |  |  | X [8] | X [3, 7] [36] | X [3, 7] |  |  | X [37] |  |  |  |  |  |  |  |  |  |  |  | X [33, 34] |  |  |
|  | **Neutrophils** | |  |  |  |  |  |  |  |  |  |  |  | X [3, 7] |  |  |  |  |  |  |  |  |  |  |  |  |  |  |  |  |  |
|  | **Mast Cells** | | X [3, 7] |  |  |  |  |  |  |  |  |  |  |  |  |  |  |  |  |  |  |  |  |  |  |  |  |  |  |  |  |
|  | **NK Cells** | |  |  |  |  |  |  |  |  |  |  | X [3, 7] | X [3, 7] |  |  |  |  |  |  |  |  |  |  |  |  |  |  |  |  |  |
|  | **Lymphocytes** | | X [6] |  |  |  |  |  |  |  |  |  | X [23] |  |  |  |  |  |  |  |  |  |  |  |  |  |  |  |  |  |  |
|  | **B cells** | |  |  |  |  |  |  |  |  |  |  |  |  |  |  |  |  |  |  |  |  |  |  |  |  |  | X [38] |  |  |  |
|  | **T cells** | |  |  |  |  |  |  | X [3, 39, 40] |  | X [3, 39, 40] |  |  |  |  |  |  |  |  |  |  |  | X [3, 39, 40] |  | X [3, 7] [3, 39, 40] |  |  |  |  |  |  |
|  | **Adipocytes** | |  |  |  |  |  |  |  |  | X [10, 11] |  | X [3, 7] |  |  |  |  |  |  |  |  |  |  |  |  |  |  |  |  |  |  |
|  | **Hepatocytes** | |  |  |  |  |  |  |  |  |  |  |  |  |  | X [3, 7] |  |  |  |  |  |  |  |  |  | X [8] | X [8] |  |  |  |  |
|  | **Platelets** | |  |  |  |  |  |  |  |  | X [10, 11] |  | X [3, 7] |  |  |  |  |  |  |  |  |  |  |  |  |  |  |  |  |  |  |
|  | **Astrocytes** | |  |  |  |  |  |  |  |  |  |  |  |  |  |  |  | X |  |  |  |  |  |  |  |  |  |  |  |  |  |
|  | **Myosatellite Cells** | |  |  |  |  |  |  |  |  |  |  |  |  |  |  |  | X |  |  |  |  |  |  |  |  |  |  |  |  |  |
|  | **Osteoclasts** | |  |  |  |  |  |  |  |  |  | X [8] |  |  |  |  |  |  |  |  |  |  |  |  |  |  |  |  |  |  |  |
|  | **Thymocytes** | |  |  |  |  |  |  |  |  |  |  |  |  |  |  |  |  |  |  | X [8] | X [8] |  |  |  |  |  |  |  |  |  |
|  | **Schwann Cells** | |  |  |  |  |  |  |  |  |  |  |  |  |  |  |  |  |  |  |  |  |  |  |  |  |  |  | X [41, 42] |  |  |
|  | **Hematopoietic Stem Cells** | |  |  |  |  |  |  |  |  |  |  |  |  |  |  |  |  |  |  | X [3, 7] |  | X [8] |  |  |  |  |  |  |  |  |
|  |  | | ***SR-A1*** | ***SR-A1.2*** | ***SR-A3*** | ***SR-A4*** | ***SR-A5*** | ***SR-A6*** | ***SR-B1*** | ***SR-B1.1*** | ***SR-B2*** | ***SR-D1*** | ***SR-E1*** | ***SR-E2*** | ***SR-E3*** | ***SR-E4*** | ***SR-F1*** | ***SR-F2*** | ***SR-F3*** | ***SR-G1*** | ***SR-H1*** | ***SR-H2*** | ***SR-I1*** | ***SR-I2*** | ***SR-I3*** | ***SR-J1*** | ***SR-J1.1*** | ***SR-K1*** | ***SR-L1*** | ***SR-L2*** |  |
| **Ligands** | **Apoptotic Cells** | | X [8] |  |  |  |  |  | X [8] |  | X [10, 11, 43, 44] |  | X [8] |  | X [3, 7] |  | X [45] | X [3, 7] |  | X [3] | X [8] | X [3, 7] |  |  |  |  |  |  | X [46] [47-49] |  |  |
|  | **AGE** | |  |  |  |  |  |  |  |  |  |  |  |  |  |  |  |  |  |  | X [8] [8, 50] | X [8] [8, 50] |  |  |  | X [4, 51] |  |  |  |  |  |
|  | **Cholesterol** | |  |  |  |  |  |  |  |  |  |  |  |  |  |  |  |  |  |  |  |  |  |  |  |  |  |  | X [33, 34] |  |  |
|  | **LPS** | | X [6] [3, 4] | X [3, 4] | X [3, 4] | X [3, 4] | X [3, 4] | X [8] [3, 4] |  |  | X [52] |  |  |  |  |  | X [45] |  |  |  |  |  |  |  |  |  |  |  |  |  |  |
|  | **LTA** | | X [3, 4] | X [3, 4] | X [3, 4] | X [3, 4] | X [3, 4] | X [3, 4] |  |  |  |  |  |  |  |  | X [45] |  |  |  |  |  |  |  |  |  |  |  |  |  |  |
|  | **LPA** | |  |  |  |  |  |  |  |  |  |  |  |  |  |  |  |  |  |  |  |  |  |  |  | X [4, 51] |  |  |  |  |  |
|  | **Bacterial CpG DNA** | | X [3, 4] | X [3, 4] | X [3, 4] | X [3, 4] | X [3, 4] | X [3, 4] |  |  |  |  |  |  |  |  |  |  |  | X [8] |  |  |  |  |  |  |  |  |  |  |  |
|  | **Gram (-) & gram (+) Bacteria** | | X [3, 7] |  |  |  | X [3, 7] | X [3, 7] |  |  | X [52] |  | X [53] [3, 7] | X [3, 7] |  |  |  |  |  |  | X [8, 50] | X [8, 50] | X [3, 7] |  |  |  |  |  |  |  |  |
|  | **Virus** | |  |  |  |  |  |  | X [8] | X [8] |  |  |  |  |  |  |  |  |  |  |  |  |  |  |  |  |  |  |  |  |  |
|  | **LDL/Modified LDL** | | X [8] [3, 4] | X [3, 4] | X [3, 4] | X [3, 4] | X [3, 4] | X [3, 4] | X [8] |  | X [10, 11, 43, 44] | X [8] [3, 7] | X [8] [23, 54] [55] |  |  |  | X [45] [56] |  |  | X [3] | X [8] | X [8] |  |  |  |  |  |  |  |  |  |
|  | **HDL/ Modified HDL** | |  |  |  |  |  |  | X [8] [3, 7] | X [8] | X [3, 7] |  |  |  |  |  |  |  |  |  |  |  |  |  |  |  |  |  |  |  |  |
|  | **C-Reactive Protein** | |  |  |  |  |  |  |  |  |  |  | X [3, 7] |  |  |  |  |  |  |  |  |  |  |  |  |  |  |  |  |  |  |
|  | **Iron/ Ferritin** | |  |  |  |  | X [8] |  |  |  |  |  |  |  |  |  |  |  |  |  |  |  |  |  |  |  |  |  |  |  |  |
|  | **Long chain fatty acids** | |  |  |  |  |  |  |  |  | X [10, 11, 43, 44] |  |  |  |  |  |  |  |  |  |  |  |  |  |  |  |  |  |  |  |  |
|  | **Thrombospondin-1** | |  |  |  |  |  |  |  |  | X [10, 11, 43, 44] |  |  |  |  |  |  |  |  |  |  |  |  |  |  |  |  |  |  |  |  |
|  | **β-amyloid (Aβ)** | | X [3, 4] | X [3, 4] | X [3, 4] | X [3, 4] | X [3, 4] | X [3, 4] |  |  | X [10, 11, 43, 44] [24] |  |  |  |  |  |  |  |  |  |  |  |  |  |  | X [4, 51] |  |  |  | X [3, 7] |  |
|  | **C1q** | |  |  |  |  |  |  |  |  |  |  |  |  |  |  | X [50, 56-58] | X [3, 7] [50, 56-58] | X [50, 56-58] |  |  |  |  |  |  | X [4, 51] |  |  | X [46] [47-49] |  |  |
|  | **Hapto-Hemoglobin** | |  |  |  |  |  |  |  |  |  |  |  |  |  |  |  |  |  |  |  |  | X [59] |  |  |  |  |  |  |  |  |
|  | **Heat Shock Proteins** | | X [3, 4] | X [3, 4] | X [3, 4] | X [3, 4] | X [3, 4] | X [3, 4] |  |  |  |  | X [8] [60-64] |  |  |  | X [45, 60-64] |  |  |  | X [60-64] |  |  |  |  |  |  |  | X [33, 34] [60-64] [34, 65] |  |  |
|  | **Ions** | |  |  |  |  |  |  |  |  |  |  |  |  |  |  |  |  |  |  |  |  |  |  |  | X [4, 51] |  |  |  | X [5] |  |
|  | **Cytokines** | |  |  |  |  |  |  |  |  |  |  |  |  |  |  |  |  |  |  |  |  |  |  |  | X [4, 51] |  |  |  |  |  |
|  | **Complex carbohydrates** | |  |  |  |  | X [8] |  |  |  |  |  |  |  |  |  |  |  |  |  |  |  |  |  |  |  |  |  |  |  |  |
|  | **Lectins** | |  |  |  |  |  |  |  |  |  | X [8] |  |  |  |  |  |  |  |  |  |  |  |  |  |  |  |  |  |  |  |
|  | **Selectins** | |  |  |  |  |  |  |  |  |  | X [8] |  |  |  |  |  |  |  |  |  |  |  |  |  |  |  |  |  |  |  |
|  | **Apolipoproteins** | |  |  |  |  |  |  |  |  |  |  |  |  |  |  | X [4] |  |  |  |  |  |  |  |  |  |  |  |  | X [5] |  |
|  | **Fungal Pathogens** | |  |  |  |  | X [8] |  |  |  | X [8] [66] |  |  | X [3, 7] |  |  | X [4] | X [3, 7] |  |  |  |  |  |  |  |  |  |  |  |  |  |
|  | **Neutrophil granule glycoproteins** | |  |  |  |  | X [8] |  |  |  |  |  |  |  |  |  |  |  |  |  |  |  |  |  |  |  |  |  |  |  |  |
|  | **Hormones** | |  |  |  |  |  |  |  |  |  |  |  |  |  |  |  |  |  |  |  |  |  |  |  |  |  |  |  | X [5] [67] |  |
|  | **HA** | |  |  |  |  |  |  |  |  |  |  |  |  |  |  |  |  |  |  |  |  |  |  |  |  |  | X 22, 23] [68] |  |  |  |
|  | **Enzymes** | |  |  |  |  |  |  |  |  |  |  |  |  |  |  |  |  |  |  |  |  |  |  |  |  |  |  |  | X [5] |  |
|  | **Calreticulin** | |  |  |  |  |  |  |  |  |  |  |  |  |  |  |  |  |  |  |  |  |  |  |  |  |  |  | X [33, 34] |  |  |
|  |  | | ***SR-A1*** | ***SR-A1.2*** | ***SR-A3*** | ***SR-A4*** | ***SR-A5*** | ***SR-A6*** | ***SR-B1*** | ***SR-B1.1*** | ***SR-B2*** | ***SR-D1*** | ***SR-E1*** | ***SR-E2*** | ***SR-E3*** | ***SR-E4*** | ***SR-F1*** | ***SR-F2*** | ***SR-F3*** | ***SR-G1*** | ***SR-H1*** | ***SR-H2*** | ***SR-I1*** | ***SR-I2*** | ***SR-I3*** | ***SR-J1*** | ***SR-J1.1*** | ***SR-K1*** | ***SR-L1*** | ***SR-L2*** |  |
| **Implicated Processes** | **Endocytosis** | |  |  |  |  | X [8] |  | X [8] |  |  | X [8] | X [23, 54] |  |  |  |  |  |  |  | X [8] |  | X [59] |  |  |  |  |  |  |  |  |
|  | **Efferocytosis** | | X |  |  |  |  | X |  |  | X [10, 11, 43] |  |  |  |  |  | X [37] |  | X |  |  |  |  |  |  |  |  |  |  |  |  |
|  | **Inflammation** | |  |  |  |  |  |  |  | X [8] |  |  | X [8] [54, 69] |  |  |  |  |  |  | X [25] | X [70] | X [70] |  |  |  | X [8] |  |  | X [71, 72] [35] |  |  |
|  | **Apoptosis** | |  |  | X [8] |  |  |  |  | X [8] |  |  | X [8] |  |  |  |  |  |  |  |  |  |  |  |  |  |  |  |  |  |  |
|  | **Angiogenesis** | |  |  |  |  |  |  |  | X [8] | [10, 11, 43] |  |  |  |  |  |  |  |  |  | X [3] | X [3] |  |  |  |  |  |  |  |  |  |
|  | **ROS Production** | |  |  | X [3, 7] |  |  |  |  |  | X [8] |  | X [8] |  |  |  |  |  |  |  |  |  |  |  |  |  |  |  |  |  |  |
|  | **Atherosclerotic Plaque Formation/ Atherogenesis** | | X [8] |  |  |  |  |  | X | X [8] | X [3, 7] [73] [74, 75] | X [8] | X [8] [23, 54] [76] |  |  |  |  |  |  |  |  |  |  |  |  |  |  |  |  |  |  |
|  | **Lipid Metabolism** | |  |  |  |  |  |  | X [77, 78] | X | [10, 11, 43] |  |  |  |  |  |  |  |  |  |  |  |  |  |  |  |  |  |  |  |  |
|  | **Cholesterol Metabolism** | |  |  |  |  |  |  | X [77, 78] | X |  |  |  |  |  |  |  |  |  |  |  |  |  |  |  |  |  |  |  |  |  |
|  | **Foam Cell Formation** | | X [8] |  |  |  |  |  | X | X [8] | X [3, 7] [73] [74, 75] | X [8] | X [55] [76] | X |  |  |  |  |  |  |  |  |  |  |  |  |  |  |  |  |  |
|  | **Phagocytosis** | | X [79] |  |  |  |  | X [79] |  |  | X [10, 11, 43] |  |  |  | X [3, 7] |  |  |  |  | X [3, 7] | X [8] | X [8] | X [59] |  |  | [22] |  |  |  |  |  |
|  | **Cytokine/ Chemokine Regulation** | |  |  |  |  |  |  |  | X [8] | X [52, 80] |  |  |  |  |  |  |  |  | X [3, 7] |  |  |  |  |  |  |  |  | X [34, 65] |  |  |
|  | **Development (embryogenesis)** | |  |  |  |  |  |  |  |  |  |  |  |  |  |  |  |  |  |  | X [8] |  |  |  |  |  |  |  |  |  |  |
|  | **Tumor progression/ regulation** | | X [8] |  | X [8] |  |  |  |  |  | X [8] |  | X [8] |  |  |  |  |  |  |  | X [8] |  | X [8] |  |  |  |  |  |  |  |  |
|  | **Cellular Development & Maintenance** | **EC** |  |  |  |  |  |  |  |  |  |  |  |  |  |  |  |  |  |  |  |  |  |  |  |  |  |  |  |  |  |
|  |  | **Smooth Muscle** |  |  |  |  |  |  |  |  |  |  |  |  |  |  |  |  |  |  |  |  |  |  |  |  |  |  |  |  |  |
|  |  | **RBC** |  |  |  |  |  |  | X [81] |  |  |  |  |  |  |  |  |  |  |  |  |  | X [8] |  |  |  |  |  |  |  |  |
|  |  | **Dendritic** |  |  |  |  |  |  |  |  |  |  |  |  |  |  |  |  |  |  |  |  |  |  |  |  |  |  |  |  |  |
|  |  | **Macrophages** |  |  |  |  |  |  |  |  |  |  |  |  |  |  |  |  |  |  |  |  |  | X [3, 7] |  |  |  |  |  |  |  |
|  |  | **Neutrophil** |  |  |  |  |  |  |  |  |  |  |  |  |  |  |  |  |  |  |  |  |  |  |  |  |  |  |  |  |  |
|  |  | **B Cells** |  |  |  |  |  |  |  |  |  |  |  |  |  |  |  |  |  |  |  |  |  |  |  |  |  |  |  |  |  |
|  |  | **T Cells** |  |  |  |  |  |  |  |  |  |  |  |  |  |  |  |  |  |  | X [82, 83] |  |  |  |  |  |  |  |  |  |  |
|  | **Migration, Recruitment, Adhesion, or Activation** | **EC** |  |  |  |  |  |  |  |  |  |  |  |  |  |  |  |  |  |  |  |  |  |  |  |  |  |  |  |  |  |
|  |  | **Smooth Muscle** |  |  |  |  |  |  |  |  |  |  |  |  |  |  |  |  |  |  |  |  |  |  |  |  |  |  |  |  |  |
|  |  | **Platelet** |  |  |  |  |  |  |  | X [8] |  |  |  |  |  |  |  |  |  |  |  |  |  |  |  |  |  |  |  |  |  |
|  |  | **RBC** |  |  |  |  |  |  |  |  |  |  |  |  |  |  |  |  |  |  |  |  |  |  |  |  |  |  |  |  |  |
|  |  | **Dendritic** |  |  |  |  |  |  |  |  | X [8] |  |  |  |  |  |  |  |  | X [8] [84] |  |  |  |  |  |  |  |  |  |  |  |
|  |  | **Macrophages** | X [85, 86] |  |  |  |  |  |  |  | X [8] [87] | X [28] |  |  |  |  |  |  |  | X [84] |  |  | X [88-91] [92] |  |  |  |  |  |  |  |  |
|  |  | **Neutrophil** | X [93] |  |  |  |  |  |  |  |  |  |  |  |  |  |  |  |  |  |  |  |  |  |  |  |  |  |  |  |  |
|  |  | **Lymphocyte** |  |  |  |  |  |  |  |  |  |  |  |  |  |  |  |  |  |  | X [3] [94] | X [3] |  |  |  |  |  |  |  |  |  |
|  |  | **NK Cells** |  |  |  |  |  |  |  |  |  |  |  |  |  |  |  |  |  | X [8] |  |  |  |  |  |  |  |  |  |  |  |
|  |  | **B Cells** |  |  |  |  |  |  |  |  |  |  |  |  |  |  |  |  |  | X [3] | X [8, 19, 56, 95] |  |  |  |  |  |  |  |  |  |  |
|  |  | **T Cells** | X [96, 97] | X [96, 97] | X [96, 97] | X [96, 97] | X [96, 97] | X [96, 97] |  |  |  |  |  |  |  |  | X [17] |  |  | X [8] [3] | X [8] [8, 19, 56, 95] |  |  |  |  |  |  | X [98] | X [34, 65] |  |  |
|  |  |  | ***SR-A1*** | ***SR-A1.2*** | ***SR-A3*** | ***SR-A4*** | ***SR-A5*** | ***SR-A6*** | ***SR-B1*** | ***SR-B1.1*** | ***SR-B2*** | ***SR-D1*** | ***SR-E1*** | ***SR-E2*** | ***SR-E3*** | ***SR-E4*** | ***SR-F1*** | ***SR-F2*** | ***SR-F3*** | ***SR-G1*** | ***SR-H1*** | ***SR-H2*** | ***SR-I1*** | ***SR-I2*** | ***SR-I3*** | ***SR-J1*** | ***SR-J1.1*** | ***SR-K1*** | ***SR-L1*** | ***SR-L2*** |  |
| **Implicated Disease** | **Autoimmune** | **SLE** | X [99, 100] |  |  |  |  | X [99, 100] |  |  | X [101, 102] [73] |  | X [76] [103] | X | X [104] |  | X [105] [37] |  |  | X [106] [107] |  |  | X [108] [92] [109] [110, 111] |  |  | X [51] | X [51] | X [112] [113] [114] [115, 116] | X [117] [49] | X [118] |  |
|  |  | **Sjorgen’s Disease** |  |  |  |  |  |  |  |  |  |  |  |  |  |  |  |  |  |  |  |  |  |  |  |  |  | X [119] |  |  |  |
|  |  | **Hashimoto’s thyroiditis** |  |  |  |  |  |  |  |  |  |  |  |  |  |  |  |  |  |  |  |  |  |  |  |  |  | X [120] |  |  |  |
|  |  | **Rheumatoid Arthritis** |  |  |  |  |  |  |  |  |  |  |  |  | X [29] [30-32] |  |  |  |  |  |  |  | X [121] |  |  | X [51] | X [51] | X [113] | X [117] | X [118] |  |
|  |  | **Multiple sclerosis (MS)** |  |  |  |  |  |  |  |  |  |  |  |  |  |  |  |  |  |  |  |  |  |  |  | X [51] | X [51] | X [113] | X [122] |  |  |
|  |  | **Diabetes (Type I)** |  |  |  |  |  |  |  |  |  |  |  |  |  |  |  |  |  |  |  |  |  |  |  | X [51] | X [51] | X [113] |  |  |  |
|  |  | **Myasthenia gravis** |  |  |  |  |  |  |  |  |  |  |  |  |  |  |  |  |  |  |  |  |  |  |  | X [51] | X [51] |  |  |  |  |
|  |  | **Systemic sclerosis** |  |  |  |  |  |  |  |  |  |  |  |  |  |  |  |  |  |  |  |  |  |  |  |  |  |  |  | X [118] |  |
|  |  | **Celiac’s Disease** |  |  |  |  |  |  |  |  | X [123] |  |  |  |  |  |  |  |  |  |  |  |  |  |  |  |  |  |  |  |  |
|  |  | **Pemphigus vulgaris (PV)** |  |  |  |  |  |  |  |  | X [40] |  |  |  |  |  |  |  |  |  |  |  | X [40] |  |  |  |  |  |  |  |  |
|  | **Inflammatory** | **Takayasu Arteritis** |  |  |  |  |  |  | X [124] |  |  |  |  |  |  |  |  |  |  |  |  |  |  |  |  |  |  |  |  |  |  |
|  |  | **Chronic Inflammation** |  |  |  |  |  |  |  |  |  |  | X [54, 69] |  | X [29] [30-32] |  |  |  |  |  |  |  | X [29] [30-32] |  |  | X [51] [4] | X [51] |  |  |  |  |
|  |  | **Chronic Pain** |  |  |  |  |  |  |  |  |  |  |  |  |  |  |  |  |  |  |  |  |  |  |  |  |  |  | X [41, 42] |  |  |
|  |  | **Osteoarthritis** |  |  |  |  |  |  |  |  |  |  |  |  |  |  |  |  |  |  |  |  | X [125] |  |  |  |  |  |  | X [118] |  |
|  |  | **Behçet's disease** |  |  |  |  |  |  |  |  |  |  |  |  |  |  |  |  |  |  |  |  |  |  |  |  |  |  |  | X [118] |  |
|  | **CVD** | **Atherothrombosis** |  |  |  |  |  |  | X [77] |  |  |  |  |  |  |  |  |  |  |  |  |  |  |  |  |  |  |  |  |  |  |
|  |  | **Myocardial infarction** |  |  |  |  |  |  | X [77] [77, 126, 127] |  |  |  |  |  |  |  |  |  |  |  |  |  |  |  |  |  |  |  |  |  |  |
|  |  | **Ischemic Cardiomyopathy** |  |  |  |  |  |  | X [77] [77, 126, 127] |  |  |  |  |  |  |  |  |  |  |  |  |  |  |  |  |  |  |  |  |  |  |
|  |  | **Atherosclerotic CVD** |  |  |  |  |  |  | X [77, 126, 127] |  | X |  | X [54, 69] [76] | X |  |  |  |  |  |  | X [70] | X [70] |  |  |  |  |  |  |  |  |  |
|  |  | **Myocardial ischemia** |  |  |  |  |  |  |  |  |  |  | X [54, 69] |  |  |  |  |  |  |  |  |  |  |  |  |  |  |  |  |  |  |
|  | **Neurodegenerative** | **Amyotrophic Lateral Sclerosis (ALS)** |  |  |  |  |  |  |  |  |  |  |  |  |  |  |  |  |  |  |  |  |  |  |  | X [51] | X [51] |  |  |  |  |
|  |  | **Alzheimer’s Disease** |  |  |  |  |  |  |  |  |  |  |  |  |  |  |  |  | X |  |  |  |  |  |  |  |  |  | X [128-130] | X |  |
|  | **Rare** | **Van den Ende-Gupta Syndrome** |  |  |  |  |  |  |  |  |  |  |  |  |  |  |  | X |  |  |  |  |  |  |  |  |  |  |  |  |  |
|  | **Chronic Liver Diseases** | **Nonalcoholic Fatty Liver Disease** |  |  |  |  |  |  | X [131] |  |  |  |  |  |  |  |  |  |  |  |  |  |  |  |  |  |  |  |  |  |  |
|  |  | **Primary sclerosing cholangitis (PSC)** |  |  |  |  |  |  |  |  |  |  |  |  |  |  | X [17] |  |  |  |  |  |  |  |  |  |  |  |  |  |  |
|  |  | **Primary biliary cholangitis (PBC)** |  |  |  |  |  |  |  |  |  |  |  |  |  |  | X [17] |  |  |  |  |  |  |  |  |  |  |  |  |  |  |
|  |  | **Alcoholic Liver Disease (ALD)** |  |  |  |  |  |  |  |  |  |  |  |  |  |  | X [17] |  |  |  |  |  |  |  |  |  |  |  |  |  |  |
|  |  | **Non-alcoholic steatohepatitis (NASH)** |  |  |  |  |  |  |  |  |  |  |  |  |  |  | X [17] |  |  |  |  |  |  |  |  |  |  |  |  |  |  |

References:

1. Patten, D.A., et al., *Scavenger Receptors: Novel Roles in the Pathogenesis of Liver Inflammation and Cancer.* Semin Liver Dis, 2022. **42**(1): p. 61-76.

2. PrabhuDas, M.R., et al., *A Consensus Definitive Classification of Scavenger Receptors and Their Roles in Health and Disease.* J Immunol, 2017. **198**(10): p. 3775-3789.

3. Alquraini, A. and J. El Khoury, *Scavenger receptors.* Curr Biol, 2020. **30**(14): p. R790-R795.

4. Taban, Q., et al., *Scavenger receptors in host defense: from functional aspects to mode of action.* Cell Commun Signal, 2022. **20**(1): p. 2.

5. Beenken, A., et al., *Structures of LRP2 reveal a molecular machine for endocytosis.* Cell, 2023. **186**(4): p. 821-836 e13.

6. Sheng, W., G. Ji, and L. Zhang, *Role of macrophage scavenger receptor MSR1 in the progression of non-alcoholic steatohepatitis.* Frontiers in Immunology, 2022. **13**.

7. Hughes, D.A., I.P. Fraser, and S. Gordon, *Murine macrophage scavenger receptor: in vivo expression and function as receptor for macrophage adhesion in lymphoid and non-lymphoid organs.* Eur J Immunol, 1995. **25**(2): p. 466-73.

8. Zani, I.A., et al., *Scavenger receptor structure and function in health and disease.* Cells, 2015. **4**(2): p. 178-201.

9. Malerød, L., et al., *The expression of scavenger receptor class B, type I (SR-BI) and caveolin-1 in parenchymal and nonparenchymal liver cells.* Cell Tissue Res, 2002. **307**(2): p. 173-80.

10. Cho, S., et al., *The Class B Scavenger Receptor CD36 Mediates Free Radical Production and Tissue Injury in Cerebral Ischemia.* The Journal of Neuroscience, 2005. **25**(10): p. 2504-2512.

11. Febbraio, M., D.P. Hajjar, and R.L. Silverstein, *CD36: a class B scavenger receptor involved in angiogenesis, atherosclerosis, inflammation, and lipid metabolism.* J Clin Invest, 2001. **108**(6): p. 785-91.

12. Duryee, M.J., et al., *Scavenger receptors on sinusoidal liver endothelial cells are involved in the uptake of aldehyde-modified proteins.* Mol Pharmacol, 2005. **68**(5): p. 1423-30.

13. Mehta, J.L., et al., *Deletion of LOX-1 Reduces Atherogenesis in LDLR Knockout Mice Fed High Cholesterol Diet.* Circulation Research, 2007. **100**(11): p. 1634-1642.

14. Wang, X., M.I. Phillips, and J.L. Mehta, *LOX-1 and Angiotensin Receptors, and Their Interplay.* Cardiovascular Drugs and Therapy, 2011. **25**(5): p. 401-417.

15. Elvevold, K., et al., *Liver sinusoidal endothelial cells depend on mannose receptor-mediated recruitment of lysosomal enzymes for normal degradation capacity.* Hepatology, 2008. **48**(6): p. 2007-15.

16. Malovic, I., et al., *The mannose receptor on murine liver sinusoidal endothelial cells is the main denatured collagen clearance receptor.* Hepatology, 2007. **45**(6): p. 1454-61.

17. Patten, D.A., et al., *SCARF-1 promotes adhesion of CD4+ T cells to human hepatic sinusoidal endothelium under conditions of shear stress.* Scientific Reports, 2017. **7**(1): p. 17600.

18. Patten, D.A., et al., *Human liver sinusoidal endothelial cells promote intracellular crawling of lymphocytes during recruitment: A new step in migration.* Hepatology, 2017. **65**(1): p. 294-309.

19. Shetty, S., et al., *Common lymphatic endothelial and vascular endothelial receptor-1 mediates the transmigration of regulatory T cells across human hepatic sinusoidal endothelium.* J Immunol, 2011. **186**(7): p. 4147-55.

20. Shetty, S., et al., *Recruitment mechanisms of primary and malignant B cells to the human liver.* Hepatology, 2012. **56**(4): p. 1521-31.

21. Jung, M.-Y., S.-Y. Park, and I.-S. Kim, *Stabilin-2 is involved in lymphocyte adhesion to the hepatic sinusoidal endothelium via the interaction with αMβ2 integrin.* Journal of Leukocyte Biology, 2007. **82**(5): p. 1156-1165.

22. Pieterse, E., et al., *Neutrophil Extracellular Traps Drive Endothelial-to-Mesenchymal Transition.* Arteriosclerosis, Thrombosis, and Vascular Biology, 2017. **37**(7): p. 1371-1379.

23. Twigg, M.W., et al., *The LOX-1 Scavenger Receptor and Its Implications in the Treatment of Vascular Disease.* Cardiol Res Pract, 2012. **2012**: p. 632408.

24. Li, X., et al., *Prostaglandin E<sub>2</sub> Receptor Subtype 2 Regulation of Scavenger Receptor CD36 Modulates Microglial A&#x3b2;<sub>42</sub> Phagocytosis.* The American Journal of Pathology, 2015. **185**(1): p. 230-239.

25. Lepore, F., et al., *CXCL16/CXCR6 Axis Drives Microglia/Macrophages Phenotype in Physiological Conditions and Plays a Crucial Role in Glioma.* Front Immunol, 2018. **9**: p. 2750.

26. Cheng, C., et al., *Recognition of lipoproteins by scavenger receptor class A members.* J Biol Chem, 2021. **297**(2): p. 100948.

27. Voloshyna, I., et al., *Cox-2-Dependent and Independent Effects of Cox-2 Inhibitors and Nsaids on Proatherogenic Changes in Human Monocytes/Macrophages.* Journal of Investigative Medicine, 2017. **65**(3): p. 694-704.

28. Ceyhan, G.O., et al., *Neural fractalkine expression is closely linked to pain and pancreatic neuritis in human chronic pancreatitis.* Laboratory Investigation, 2009. **89**(3): p. 347-361.

29. Hanlon, M.M., et al., *Loss of synovial tissue macrophage homeostasis precedes rheumatoid arthritis clinical onset.* Science Advances, 2024. **10**(39): p. eadj1252.

30. Román-Fernández, I.V., et al., *Assessment of CD40 and CD40L expression in rheumatoid arthritis patients, association with clinical features and DAS28.* Clin Exp Med, 2019. **19**(4): p. 427-437.

31. Kawai, T., et al., *Thromboembolic complications after treatment with monoclonal antibody against CD40 ligand.* Nature Medicine, 2000. **6**(2): p. 114-114.

32. Suttles, J. and R.D. Stout, *Macrophage CD40 signaling: a pivotal regulator of disease protection and pathogenesis.* Semin Immunol, 2009. **21**(5): p. 257-64.

33. Basu, S., et al., *CD91 is a common receptor for heat shock proteins gp96, hsp90, hsp70, and calreticulin.* Immunity, 2001. **14**(3): p. 303-13.

34. Binder, R.J., et al., *CD91-Dependent Modulation of Immune Responses by Heat Shock Proteins: A Role in Autoimmunity.* Autoimmune Dis, 2012. **2012**: p. 863041.

35. Yancey, P.G., et al., *Macrophage LRP-1 controls plaque cellularity by regulating efferocytosis and Akt activation.* Arterioscler Thromb Vasc Biol, 2010. **30**(4): p. 787-95.

36. Lood, C., et al., *C1q inhibits immune complex-induced interferon-alpha production in plasmacytoid dendritic cells: a novel link between C1q deficiency and systemic lupus erythematosus pathogenesis.* Arthritis Rheum, 2009. **60**(10): p. 3081-90.

37. Jorge, A.M., et al., *SCARF1-Induced Efferocytosis Plays an Immunomodulatory Role in Humans, and Autoantibodies Targeting SCARF1 Are Produced in Patients with Systemic Lupus Erythematosus.* J Immunol, 2022. **208**(4): p. 955-967.

38. Yi, P., et al., *Overexpressed CD44 is associated with B-cell activation via the HA-CD44-AIM2 pathway in lupus B cells.* Clin Immunol, 2023. **255**: p. 109710.

39. Rueda, C.M., et al., *High density lipoproteins selectively promote the survival of human regulatory T cells.* Journal of Lipid Research, 2017. **58**(8): p. 1514-1523.

40. Das, D., et al., *T helper type 1 polarizing γδ T cells and Scavenger receptors contribute to the pathogenesis of Pemphigus vulgaris.* Immunology, 2018. **153**(1): p. 97-104.

41. Orita, S., et al., *Schwann cell LRP1 regulates remak bundle ultrastructure and axonal interactions to prevent neuropathic pain.* J Neurosci, 2013. **33**(13): p. 5590-602.

42. Poplawski, G., et al., *Schwann cells regulate sensory neuron gene expression before and after peripheral nerve injury.* Glia, 2018. **66**(8): p. 1577-1590.

43. Hirano, K., et al., *Pathophysiology of human genetic CD36 deficiency.* Trends Cardiovasc Med, 2003. **13**(4): p. 136-41.

44. Park, L., et al., *Scavenger receptor CD36 is essential for the cerebrovascular oxidative stress and neurovascular dysfunction induced by amyloid-beta.* Proc Natl Acad Sci U S A, 2011. **108**(12): p. 5063-8.

45. Patten, D.A., *SCARF1: a multifaceted, yet largely understudied, scavenger receptor.* Inflamm Res, 2018. **67**(8): p. 627-632.

46. Byrne, J.C., et al., *Bruton's tyrosine kinase is required for apoptotic cell uptake via regulating the phosphorylation and localization of calreticulin.* J Immunol, 2013. **190**(10): p. 5207-15.

47. Duus, K., et al., *Direct interaction between CD91 and C1q.* Febs j, 2010. **277**(17): p. 3526-37.

48. Mikołajczyk, T.P., et al., *Characterization of the impairment of the uptake of apoptotic polymorphonuclear cells by monocyte subpopulations in systemic lupus erythematosus.* Lupus, 2014. **23**(13): p. 1358-1369.

49. Donnelly, S., et al., *Impaired recognition of apoptotic neutrophils by the C1q/calreticulin and CD91 pathway in systemic lupus erythematosus.* Arthritis Rheum, 2006. **54**(5): p. 1543-56.

50. Ishii, J., et al., *SREC-II, a new member of the scavenger receptor type F family, trans-interacts with SREC-I through its extracellular domain.* J Biol Chem, 2002. **277**(42): p. 39696-702.

51. Dong, H., et al., *Pathophysiology of RAGE in inflammatory diseases.* Front Immunol, 2022. **13**: p. 931473.

52. Hoebe, K., et al., *CD36 is a sensor of diacylglycerides.* Nature, 2005. **433**(7025): p. 523-7.

53. Korkmaz, F.T., et al., *Lectin-like oxidized low-density lipoprotein receptor 1 attenuates pneumonia-induced lung injury.* JCI Insight, 2022. **7**(23).

54. Pyrpyris, N., et al., *LOX-1 Receptor: A Diagnostic Tool and Therapeutic Target in Atherogenesis.* Current Problems in Cardiology, 2024. **49**(1, Part C): p. 102117.

55. Kattoor, A.J., A. Goel, and J.L. Mehta, *LOX-1: Regulation, Signaling and Its Role in Atherosclerosis.* Antioxidants (Basel), 2019. **8**(7).

56. Adachi, H., et al., *Expression cloning of a novel scavenger receptor from human endothelial cells.* J Biol Chem, 1997. **272**(50): p. 31217-20.

57. Wicker-Planquart, C., et al., *Insights into the ligand binding specificity of SREC-II (scavenger receptor expressed by endothelial cells).* FEBS Open Bio, 2021. **11**(10): p. 2693-2704.

58. Iram, T., et al., *Megf10 Is a Receptor for C1Q That Mediates Clearance of Apoptotic Cells by Astrocytes.* J Neurosci, 2016. **36**(19): p. 5185-92.

59. Kristiansen, M., et al., *Identification of the haemoglobin scavenger receptor.* Nature, 2001. **409**(6817): p. 198-201.

60. Thériault, J.R., H. Adachi, and S.K. Calderwood, *Role of scavenger receptors in the binding and internalization of heat shock protein 70.* J Immunol, 2006. **177**(12): p. 8604-11.

61. Calderwood, S.K., et al., *Cell surface receptors for molecular chaperones.* Methods, 2007. **43**(3): p. 199-206.

62. Jacquemin, C., et al., *Heat shock protein 70 potentiates interferon alpha production by plasmacytoid dendritic cells: relevance for cutaneous lupus and vitiligo pathogenesis.* British Journal of Dermatology, 2017. **177**(5): p. 1367-1375.

63. Berwin, B., et al., *SREC-I, a Type F Scavenger Receptor, Is an Endocytic Receptor for Calreticulin*.* Journal of Biological Chemistry, 2004. **279**(49): p. 51250-51257.

64. Facciponte, J.G., X.Y. Wang, and J.R. Subjeck, *Hsp110 and Grp170, members of the Hsp70 superfamily, bind to scavenger receptor-A and scavenger receptor expressed by endothelial cells-I.* Eur J Immunol, 2007. **37**(8): p. 2268-79.

65. Binder, R.J. and P.K. Srivastava, *Essential role of CD91 in re-presentation of gp96-chaperoned peptides.* Proceedings of the National Academy of Sciences, 2004. **101**(16): p. 6128-6133.

66. Means, T.K., et al., *Evolutionarily conserved recognition and innate immunity to fungal pathogens by the scavenger receptors SCARF1 and CD36.* J Exp Med, 2009. **206**(3): p. 637-53.

67. Dvanajscak, Z., et al., *Anti-Brush Border Antibody Disease (Anti-LRP2 Nephropathy) Associated With Lupus Nephritis.* Kidney Int Rep, 2020. **5**(9): p. 1590-1594.

68. Nagy, N., et al., *Hyaluronan levels are increased systemically in human type 2 but not type 1 diabetes independently of glycemic control.* Matrix Biol, 2019. **80**: p. 46-58.

69. Thakkar, S., et al., *Structure-based Design Targeted at LOX-1, a Receptor for Oxidized Low-Density Lipoprotein.* Scientific Reports, 2015. **5**(1): p. 16740.

70. Manta, C.P., et al., *Targeting of Scavenger Receptors Stabilin-1 and Stabilin-2 Ameliorates Atherosclerosis by a Plasma Proteome Switch Mediating Monocyte/Macrophage Suppression.* Circulation, 2022. **146**(23): p. 1783-1799.

71. Overton, C.D., et al., *Deletion of macrophage LDL receptor-related protein increases atherogenesis in the mouse.* Circ Res, 2007. **100**(5): p. 670-7.

72. Gaultier, A., et al., *Regulation of tumor necrosis factor receptor-1 and the IKK-NF-kappaB pathway by LDL receptor-related protein explains the antiinflammatory activity of this receptor.* Blood, 2008. **111**(11): p. 5316-25.

73. Reiss, A.B., et al., *Enhanced CD36 scavenger receptor expression in THP-1 human monocytes in the presence of lupus plasma: linking autoimmunity and atherosclerosis.* Exp Biol Med (Maywood), 2009. **234**(3): p. 354-60.

74. Febbraio, M., et al., *Targeted disruption of the class B scavenger receptor CD36 protects against atherosclerotic lesion development in mice.* J Clin Invest, 2000. **105**(8): p. 1049-56.

75. Kuchibhotla, S., et al., *Absence of CD36 protects against atherosclerosis in ApoE knock-out mice with no additional protection provided by absence of scavenger receptor A I/II.* Cardiovasc Res, 2008. **78**(1): p. 185-96.

76. Sagar, D., et al., *LOX-1: A potential driver of cardiovascular risk in SLE patients.* PLoS One, 2020. **15**(3): p. e0229184.

77. Pearson, J.T., et al., *Widespread Coronary Dysfunction in the Absence of HDL Receptor SR-B1 in an Ischemic Cardiomyopathy Mouse Model.* Scientific Reports, 2017. **7**(1): p. 18108.

78. Staršíchová, A., *SR-B1-/-ApoE-R61h/h Mice Mimic Human Coronary Heart Disease.* Cardiovascular Drugs and Therapy, 2023.

79. Platt, N., et al., *Role for the class A macrophage scavenger receptor in the phagocytosis of apoptotic thymocytes in vitro.* Proc Natl Acad Sci U S A, 1996. **93**(22): p. 12456-60.

80. Hoebe, K., E. Janssen, and B. Beutler, *The interface between innate and adaptive immunity.* Nat Immunol, 2004. **5**(10): p. 971-4.

81. Feng, H., et al., *Deficiency of scavenger receptor BI leads to impaired lymphocyte homeostasis and autoimmune disorders in mice.* Arterioscler Thromb Vasc Biol, 2011. **31**(11): p. 2543-51.

82. Burgdorf, S., et al., *Distinct pathways of antigen uptake and intracellular routing in CD4 and CD8 T cell activation.* Science, 2007. **316**(5824): p. 612-6.

83. Carambia, A., et al., *TGF-β-dependent induction of CD4⁺CD25⁺Foxp3⁺ Tregs by liver sinusoidal endothelial cells.* J Hepatol, 2014. **61**(3): p. 594-9.

84. Korbecki, J., et al., *The Role of CXCL16 in the Pathogenesis of Cancer and Other Diseases.* Int J Mol Sci, 2021. **22**(7).

85. Robbins, C.S., et al., *Local proliferation dominates lesional macrophage accumulation in atherosclerosis.* Nat Med, 2013. **19**(9): p. 1166-72.

86. Tabas, I. and K.E. Bornfeldt, *Macrophage Phenotype and Function in Different Stages of Atherosclerosis.* Circ Res, 2016. **118**(4): p. 653-67.

87. Park, Y.M., M. Febbraio, and R.L. Silverstein, *CD36 modulates migration of mouse and human macrophages in response to oxidized LDL and may contribute to macrophage trapping in the arterial intima.* J Clin Invest, 2009. **119**(1): p. 136-45.

88. Cheng, Q., et al., *Ferroptosis of CD163+ tissue-infiltrating macrophages and CD10+ PC+ epithelial cells in lupus nephritis.* Frontiers in Immunology, 2023. **14**.

89. Endo, N., et al., *Urinary soluble CD163 level reflects glomerular inflammation in human lupus nephritis.* Nephrology Dialysis Transplantation, 2016. **31**(12): p. 2023-2033.

90. Moestrup, S.K. and H.J. Møller, *CD163: a regulated hemoglobin scavenger receptor with a role in the anti-inflammatory response.* Ann Med, 2004. **36**(5): p. 347-54.

91. Skytthe, M.K., J.H. Graversen, and S.K. Moestrup, *Targeting of CD163(+) Macrophages in Inflammatory and Malignant Diseases.* Int J Mol Sci, 2020. **21**(15).

92. Crayne, C.B., et al., *The Immunology of Macrophage Activation Syndrome.* Frontiers in Immunology, 2019. **10**.

93. Zhu, Y., et al., *Stimulation of the class-A scavenger receptor induces neutrophil extracellular traps (NETs) by ERK dependent NOX2 and ROMO1 activation.* Biochemical and Biophysical Research Communications, 2019. **511**(4): p. 847-854.

94. Irjala, H., et al., *The same endothelial receptor controls lymphocyte traffic both in vascular and lymphatic vessels.* Eur J Immunol, 2003. **33**(3): p. 815-24.

95. Karikoski, M., et al., *Clever-1/Stabilin-1 regulates lymphocyte migration within lymphatics and leukocyte entrance to sites of inflammation.* Eur J Immunol, 2009. **39**(12): p. 3477-87.

96. Xu, Z., et al., *Innate scavenger receptor-A regulates adaptive T helper cell responses to pathogen infection.* Nature Communications, 2017. **8**(1): p. 16035.

97. Zuo, D., et al., *Scavenger receptor A restrains T-cell activation and protects against concanavalin A-induced hepatic injury.* Hepatology, 2013. **57**(1): p. 228-38.

98. Jordan, A.R., et al., *The Role of CD44 in Disease Pathophysiology and Targeted Treatment.* Front Immunol, 2015. **6**: p. 182.

99. Chen, X.W., et al., *Anti-class a scavenger receptor autoantibodies from systemic lupus erythematosus patients impair phagocytic clearance of apoptotic cells by macrophages in vitro.* Arthritis Res Ther, 2011. **13**(1): p. R9.

100. Wermeling, F., et al., *Class A scavenger receptors regulate tolerance against apoptotic cells, and autoantibodies against these receptors are predictive of systemic lupus.* J Exp Med, 2007. **204**(10): p. 2259-65.

101. Tabas, I., *Macrophage death and defective inflammation resolution in atherosclerosis.* Nature Reviews Immunology, 2010. **10**(1): p. 36-46.

102. Tabas, I., G. García-Cardeña, and G.K. Owens, *Recent insights into the cellular biology of atherosclerosis.* Journal of Cell Biology, 2015. **209**(1): p. 13-22.

103. Li, D., et al., *C-type lectin receptor Dectin3 deficiency balances the accumulation and function of FoxO1-mediated LOX-1(+) M-MDSCs in relieving lupus-like symptoms.* Cell Death Dis, 2021. **12**(9): p. 829.

104. Gordon, S., *Pattern recognition receptors: doubling up for the innate immune response.* Cell, 2002. **111**(7): p. 927-30.

105. Ramirez-Ortiz, Z.G., et al., *The scavenger receptor SCARF1 mediates the clearance of apoptotic cells and prevents autoimmunity.* Nat Immunol, 2013. **14**(9): p. 917-26.

106. Qin, M., et al., *Elevated levels of serum sCXCL16 in systemic lupus erythematosus; potential involvement in cutaneous and renal manifestations.* Clin Rheumatol, 2014. **33**(11): p. 1595-601.

107. Hassan, A.M., et al., *Serum-soluble CXCL16 in juvenile systemic lupus erythematosus: a promising predictor of disease severity and lupus nephritis.* Clin Rheumatol, 2018. **37**(11): p. 3025-3032.

108. Huang, Y.J., et al., *Urine Soluble CD163 Is a Promising Biomarker for the Diagnosis and Evaluation of Lupus Nephritis.* Front Immunol, 2022. **13**: p. 935700.

109. Mukherjee, R., et al., *Non-Classical monocytes display inflammatory features: Validation in Sepsis and Systemic Lupus Erythematous.* Scientific Reports, 2015. **5**(1): p. 13886.

110. Olmes, G., et al., *CD163+ M2c-like macrophages predominate in renal biopsies from patients with lupus nephritis.* Arthritis Res Ther, 2016. **18**: p. 90.

111. Li, J., et al., *Significance of CD163-Positive Macrophages in Proliferative Glomerulonephritis.* Am J Med Sci, 2015. **350**(5): p. 387-92.

112. Yung, S. and T.M. Chan, *The Role of Hyaluronan and CD44 in the Pathogenesis of Lupus Nephritis.* Autoimmune Dis, 2012. **2012**: p. 207190.

113. Yoshioka, Y., et al., *Suppression of hyaluronan synthesis alleviates inflammatory responses in murine arthritis and in human rheumatoid synovial fibroblasts.* Arthritis Rheum, 2013. **65**(5): p. 1160-70.

114. Saeed, M., *Novel linkage disequilibrium clustering algorithm identifies new lupus genes on meta-analysis of GWAS datasets.* Immunogenetics, 2017. **69**(5): p. 295-302.

115. Li, Y., et al., *Phosphorylated ERM is responsible for increased T cell polarization, adhesion, and migration in patients with systemic lupus erythematosus.* J Immunol, 2007. **178**(3): p. 1938-47.

116. Crispin, J.C., et al., *Expression of CD44 variant isoforms CD44v3 and CD44v6 is increased on T cells from patients with systemic lupus erythematosus and is correlated with disease activity.* Arthritis Rheum, 2010. **62**(5): p. 1431-7.

117. Gorovoy, M., et al., *Inflammatory mediators promote production of shed LRP1/CD91, which regulates cell signaling and cytokine expression by macrophages.* J Leukoc Biol, 2010. **88**(4): p. 769-78.

118. Ooka, S., et al., *Autoantibodies to low-density-lipoprotein-receptor-related protein 2 (LRP2) in systemic autoimmune diseases.* Arthritis Res Ther, 2003. **5**(3): p. R174-80.

119. Tishler, M., et al., *Salivary and serum hyaluronic acid concentrations in patients with Sjögren’s syndrome.* Annals of the Rheumatic Diseases, 1998. **57**(8): p. 506-506.

120. Gianoukakis, A.G., et al., *Hyaluronan accumulation in thyroid tissue: evidence for contributions from epithelial cells and fibroblasts.* Endocrinology, 2007. **148**(1): p. 54-62.

121. Greisen, S.R., et al., *Soluble macrophage-derived CD163 is a marker of disease activity and progression in early rheumatoid arthritis.* Clin Exp Rheumatol, 2011. **29**(4): p. 689-92.

122. Pazhouhandeh, M., et al., *A systems medicine approach reveals disordered immune system and lipid metabolism in multiple sclerosis patients.* Clin Exp Immunol, 2018. **192**(1): p. 18-32.

123. Cupi, M.L., et al., *Defective Expression of Scavenger Receptors in Celiac Disease Mucosa.* PLOS ONE, 2014. **9**(6): p. e100980.

124. Mutoh, T., et al., *Identification of two major autoantigens negatively regulating endothelial activation in Takayasu arteritis.* Nat Commun, 2020. **11**(1): p. 1253.

125. Ohashi, Y., et al., *Correlation between CD163 expression and resting pain in patients with hip osteoarthritis: Possible contribution of CD163+ monocytes/macrophages to pain pathogenesis.* Journal of Orthopaedic Research, 2022. **40**(6): p. 1365-1374.

126. Zhang, S., et al., *Diet-induced occlusive coronary atherosclerosis, myocardial infarction, cardiac dysfunction, and premature death in scavenger receptor class B type I-deficient, hypomorphic apolipoprotein ER61 mice.* Circulation, 2005. **111**(25): p. 3457-64.

127. Nakaoka, H., et al., *Establishment of a novel murine model of ischemic cardiomyopathy with multiple diffuse coronary lesions.* PLoS One, 2013. **8**(8): p. e70755.

128. García-Fernández, P., N. Üçeyler, and C. Sommer, *From the low-density lipoprotein receptor-related protein 1 to neuropathic pain: a potentially novel target.* Pain Rep, 2021. **6**(1): p. e898.

129. Gaultier, A., et al., *A shed form of LDL receptor-related protein-1 regulates peripheral nerve injury and neuropathic pain in rodents.* J Clin Invest, 2008. **118**(1): p. 161-72.

130. Patel, P. and J. Shah, *Role of Vitamin D in Amyloid clearance via LRP-1 upregulation in Alzheimer's disease: A potential therapeutic target?* J Chem Neuroanat, 2017. **85**: p. 36-42.

131. Tarantino, G., V. Citro, and D. Capone, *Nonalcoholic Fatty Liver Disease: A Challenge from Mechanisms to Therapy.* J Clin Med, 2019. **9**(1).
